# Variability in Primary Care Telehealth Delivery Methods Across Chronic Conditions

**DOI:** 10.1001/jamanetworkopen.2025.1988

**Published:** 2025-03-26

**Authors:** Jacqueline M. Ferguson, Liberty Greene, James Van Campen, Donna M. Zulman, Charlie M. Wray

**Affiliations:** 1 Center for Innovation to Implementation, Veterans Affairs Palo Alto Health Care System, Menlo Park, California; 2 Division of Primary Care and Population Health, Stanford University School of Medicine, Stanford, California; 3 Department of Medicine, University of California San Francisco; 4 Section of Hospital Medicine, Veterans Affairs San Francisco Health Care System, San Francisco, California

## Abstract

**Importance:**

Sociodemographic characteristics, such as age, gender, and urbanicity, are associated individual’s engagement with telehealth. However, it is unknown whether an individual’s specific clinical conditions (eg, hypertension, low back pain, or heart failure) are associated with the likelihood of receiving in person, telephone, or video-based care.

**Objective:**

To identify chronic conditions that are more or less likely to be managed in person, via telephone, or via video.

**Design, Setting, and Participants:**

This cross-sectional study used data from nationwide Veterans Health Administration (VHA) patient electronic health records between April 1, 2022, and March 31, 2023.

**Exposure:**

Thirty-nine chronic conditions managed at each encounter were identified using *International Statistical Classification of Diseases and Related Health Problems, Tenth Revision* diagnosis codes.

**Main Outcome and Measures:**

The primary outcome was the mean probability of an encounter being completed in person, via telephone, or via video, based on the diagnosis codes for that encounter. Analyses were adjusted for sociodemographic characteristics associated with care delivery methods and other chronic conditions managed during the encounter.

**Results:**

Of the included 3 975 328 US veterans with 7 144 371 outpatient primary care encounters, 1 203 436 were aged 45 to 64 years (30.3%), 3 582 876 were male (90.1%), 382 885 were female (9.6%), 736 960 were Black or African American veterans (18.5%), and 2 566 175 were White veterans (64.6%). Among 7 144 371 outpatient primary care encounters, 83.2% (95% CI, 83.1%-84.3%) occurred in person, 11.1% (95% CI, 11.0%-11.1%) occurred via telephone, and 5.8% (95% CI, 5.77%-5.82%) occurred via video. Conditions with the highest adjusted percentage of video-based care included dementia (10.7%; 95% CI, 10.39%-10.93%), HIV or AIDS (8.8%; 95% CI, 8.41%-9.18%), pressure and chronic ulcers (8.0%; 95% CI, 7.58%-8.56%), and Parkinson disease (7.5%; 95% CI, 7.20%-7.75%). A total of 21 conditions were less likely to be managed via telephone and video when the condition was documented compared with encounters without the condition documented, including ischemic heart disease, chronic obstructive pulmonary disease, atrial fibrillation, cancer, asthma, and deafness and hearing impairment. Only 3 conditions, heart failure, HIV or AIDS, and mobility impairment, were more likely to occur via telehealth (both telephone and video) when documented compared with not documented, suggesting increased use of telehealth for these conditions.

**Conclusions and Relevance:**

The findings of this cross-sectional study suggest an individual’s chronic condition may be associated with whether they received care in person or through telehealth. The need for a physical examination, laboratory, or other diagnostic information, and the acuity of the condition may be potential drivers of this differential use. The percentage of care delivered via telephone or video may be a valuable measure to identify the optimal mix of telehealth and in-person care for various clinical scenarios.

## Key Points

**Question:**

Are specific clinical conditions managed in a primary care setting more likely to have visits completed using telehealth?

**Findings:**

In this cross-sectional study of 7 144 371 outpatient primary care encounters of 3 975 328 US veterans, visits for chronic conditions dependent on physical examination or the need for laboratory assessment were less likely to have been performed via telehealth (either via telephone or video). Individuals seen for conditions such as pressure and chronic ulcers, dementia, and Parkinson disease, which are comorbidities often associated with limited mobility and limited functional abilities, were more likely to be video based than other chronic conditions.

**Meaning:**

These findings suggest that the clinical conditions being addressed during a primary care visit may be associated with the probability of that encounter being in person, telephone-based, or video-based.

## Teaser

This cross-sectional study examines chronic conditions that are more or less likely to be managed in person or via telehealth (ie, telephone or video) using nationwide data from the Veterans Health Administration.

## Introduction

In recent years, the use of telehealth (ie, virtual care provided through telephone and video-based platforms) has increased substantially. Spurred by the COVID-19 pandemic, health care systems converted large amounts of their services to telehealth platforms. Telehealth has become a pivotal tool in health care delivery as it increases access to care for those with geographic, financial, and logistical barriers to care.^1,2^ Recent studies have found that approximately one-third of all primary care is now delivered through telehealth and suggest steady use with 43% of adults stating they’ve used telehealth in 2022.^3,4^

Well known drivers of increased telehealth use include patient characteristics (eg, younger age, female gender, urban dwelling), medical facility characteristics (eg, broadband and equipment availability), and physician preferences and support for video care (eg, belief and trust in video care).^5,6,7,8,9,10^ However, there is limited research examining which types of clinical conditions (eg, hypertension, low back pain, or heart failure) are being treated using telephone or video-based care.^11^ Understanding the distribution of telehealth across clinical conditions could enhance evaluations aimed at identifying where telehealth is over- and underused, to understand why telehealth is used in some clinical situations and not others, and to help guide health care systems as they expand their telehealth offerings.

Using national electronic Veterans Health Administration (VHA) health care record data, we identified how much outpatient primary care is being provided through telephone and video and calculated the adjusted mean probability that an encounter will be offered via telephone or video depending on the encounter diagnosis.

## Methods

This cross-sectional study followed the Strengthening the Reporting of Observational Studies in Epidemiology (STROBE) reporting guideline. This evaluation was designated as nonresearch quality improvement by VHA program office partners in the US Department of Veterans Affairs Office of Connected Care. The institutional review board at the Stanford Research Compliance Office determined this evaluation does not meet the requirement of research or clinical investigation and informed consent was waived.

### Data Sources and Study Population

Veteran patient data and outpatient encounter history were extracted from the VHA electronic health records.^12^ We examined in person, video, or telephone outpatient primary care encounters occurring nationwide within VHA between April 1, 2022, and March 31, 2023. This time period was chosen as it represents when telehealth rates stabilized following the acute phases of the COVID-19 pandemic.^4^

We defined in person, video, or telephone primary care encounters provided by primary care clinicians (eg, including physicians, nurses, but excluding social workers, mental health clinicians) using VHA Managerial Cost Accounting Stop Codes (eTable 1 in Supplement 1). We only included visits where minimal clinician decision-making was used (ie, brief telephone encounters to disclose test results) by restricting to evaluation and management encounters where a comprehensive assessment and management plan was enacted (eTable 2 in Supplement 1). We excluded 5 VHA facilities that had transitioned to the new electronic health care record system by March 31, 2022, and excluded encounters among veterans who were missing data on drive time to nearest primary care clinician or rurality of home (5% of data). We also excluded encounters corresponding to veterans for whom we had very small numbers for; including veterans who were older than 100 years old, or veterans with a drive time of greater than 5 hours (less than 726 veterans and <0.1% of data). Our analytic dataset included 3 975 328 veterans (eFigure in Supplement 1).

### Chronic Conditions Associated With Each Encounter

We defined the chronic conditions managed at each in person, video, or telephone encounter based on groupings of *International Statistical Classification of Diseases and Related Health Problems, Tenth Revision *(*ICD-10*) diagnoses codes (eTable 3 in Supplement 1) using definitions of 39 common chronic and disabling conditions treated among veterans at VHA in primary care. Definitions were derived from the Center for Medicare and Medicaid Services (CMS) Chronic Condition Warehouse (CCW)^13^ and from Healthcare Cost and Utilization Project Clinical Classification Software Refined (CCSR). We also excluded encounters that were none of the 39 conditions. Our final analytic cohort included 7 144 371 outpatient primary care encounters (eFigure in Supplement 1).

### Outcome

Our primary outcome was whether an encounter was completed via telephone or video. While it is largely believed that video-based encounters provide higher fidelity data and interactions and are thus the preferred modality within the VHA and other health care systems,^14,15,16^ telephone-based interactions are more prominent and accessible than video-based encounters, especially for individuals who lack broadband or a video-capable device.^17,18^ Our secondary outcome was whether an encounter was completed in person, providing the complement to care provided via telephone or video. We classified each encounter by care modality (in person, video, telephone) as defined previously (eTable 1 in Supplement 1).^4,7,19,20^ For each chronic condition, we calculated the percentage of telephone, video, or in-person encounters relative to all encounters. For example, the percentage of video care was calculated as the number of video encounters divided by the sum of all encounters (in person, telephone, and video). As encounters could have multiple chronic conditions coded, some encounters were counted more than once across chronic conditions.

### Veteran and Facility Characteristics Associated With Each Encounter

We included veteran characteristics, such as age, race, ethnicity, gender, rurality of the veterans’ home, and marital status. We also included VHA Enrollment Priority Group and drive time to nearest VHA primary care facility, as both are associated with VHA health care use.^19^ Race and ethnicity were the most frequent self-identified classification in veteran patient health records (American Indian or Alaska Native, Asian, Black or African American, Hispanic or Latino, more than 1 race, Native Hawaiian or Other Pacific Islander, White, or Unknown). Missing data on race, ethnicity, or marital status were treated as distinct categories. Gender was defined using a combination of self-identified gender identity (eg, man, women, nonbinary, transgender woman, transgender man) and the birth sex variable in patient health records. These data were combined using an established algorithm from the VHA Office of Health Equity to generate a gender variable with categories of man, woman, and gender diverse. Veterans’ home addresses were classified into urban, rural, and highly rural designations based on home zip code.^21^ We incorporated information from VHA’s priority-based enrollment system, which categorizes veterans into 8 groups based on their disability rating, income, recent military service, and other factors.^21^ Veterans who qualify for more than 1 priority group are assigned to the highest priority group (1 being the highest priority group).

We also adjusted for the complexity of a VHA medical center where the encounter occurred, as previous work has suggested differential use of telehealth between VHA facilities.^8^ The Facility Complexity Model classifies VHA facilities at levels 1A, 1B, 1C, 2, or 3, with level 1A being the most complex and level 3 being the least complex based on patient volume, and prevalence of complex clinical programs, and teaching and research activities.^22,23^

### Statistical Analysis

We tabulated the frequency of the 39 chronic conditions coded in an outpatient primary care encounter by care modality (ie, how frequently a condition was coded in in person, telephone, or video encounters). We also tabulated the frequency of in person, telephone- and video-based outpatient primary care encounters by veteran sociodemographic characteristics (ie, how many video encounters occurred among veterans aged 18 to 44 years). We also report the unadjusted percentage of care completed in person, via telephone, and via video for each encounter diagnosis.

Using 3 generalized linear models with a Poisson distribution with a log link, we modeled the likelihood that an encounter with a specific chronic condition listed in the diagnoses would be conducted (1) in person, (2) via telephone, or (3) via video. For each model, we report our primary results as estimative margins (eg, the probability of an encounter occurring via video when a diagnosis is coded) and as marginal effect estimates (eg, the difference in the probability of an encounter occurring via video when a diagnosis is coded vs when it is not coded). We also report risk ratios comparing when a diagnosis is coded and not coded for video (eTable 5 in Supplement 1), phone (eTable 6 in Supplement 1) and in person (eTable 7 in Supplement 1).

All models were adjusted for veteran sociodemographic characteristics known to be associated with care delivery method (eg, age, gender, race, ethnicity, rurality, marital status, VHA enrollment priority group, drive time from a primary care facility, facility complexity) and other chronic conditions managed during the encounter. The standard errors for all models were clustered at the individual patient level. We flexibly modeled age and drive time using restricted cubic splines. As we mutually adjusted for the 39 conditions in our model, we interpret the estimates for each chronic condition as an independent association.

All statistical analyses were conducted in Stata version 18 (StataCorp). Data were analyzed from October 2022 to December 2024. Statistical significance was set at *P* < .05, and all tests were 2-sided.

## Results

The 7 144 371 outpatient primary care encounters represent 3 975 328 veterans (eTable 4 in Supplement 1). Of the veterans, 1 203 436 were aged 45 to 64 years (30.3%), 3 582 876 were male (90.1%), 382 885 were female (9.6%), 736 960 were Black or African American veterans (18.5%), and 2 566 175 were White veterans (64.6%). Most were urban dwelling, married, and living within 17 to 25 minutes of a VHA primary care site. Of the total number of participants, the 5 most frequently coded chronic conditions during a primary care encounter were hypertension (3 727 795 participants [52.2%]); hyperlipidemia (3 145 307 participants [44.0%]); diabetes (1 991 392 participants [27.9%]); anxiety, depression, or posttraumatic stress disorder (PTSD) (1 425 636 participants [20.0%]); and lower back pain (1 214 558 participants [17.0%]) (Table). While the order varied, these conditions were all in the top 5 for in person, telephone, and video care (Table).

**Table.** Care Modalities of the 7 144 371 Outpatient Primary Care Encounters Occurring in the Veterans Health Administration (VHA) Between April 1, 2022, and March 31, 2023, by Diagnosis

Diagnosis	Encounters, No. (%)^a^			
	Total (n = 7 144 371)	In-Person (n = 5 940 520)	Telephone (n = 789 795)	Video (n = 414 056)
Hypertension	3 727 795 (52.2)	3 272 792 (55.1)	281 690 (35.7)	173 313 (41.9)
Hyperlipidemia	3 145 307 (44.0)	2 786 069 (46.9)	217 300 (27.5)	141 938 (34.3)
Diabetes	1 991 392 (27.9)	1 711 353 (28.8)	189 636 (24.0)	90 403 (21.8)
Anxiety, depression, PTSD	1 425 636 (20.0)	1 200 439 (20.2)	121 568 (15.4)	103 629 (25.0)
Lower back pain	1 214 558 (17.0)	1 015 140 (17.1)	120 487 (15.3)	78 931 (19.1)
Ischemic heart disease	771 811 (10.8)	687 390 (11.6)	57 623 (7.3)	26 798 (6.5)
Obesity	679 258 (9.5)	598 430 (10.1)	42 336 (5.4)	38 492 (9.3)
Benign prostatic hyperplasia	621 610 (8.7)	555 892 (9.4)	43 633 (5.5)	22 085 (5.3)
Rheumatoid arthritis or osteoarthritis	622 697 (8.7)	542 840 (9.1)	51 728 (6.5)	28 129 (6.8)
COPD	553 826 (7.8)	481 004 (8.1)	52 744 (6.7)	20 078 (4.8)
Chronic kidney disease	537 397 (7.5)	465 445 (7.8)	50 729 (6.4)	21 223 (5.1)
Fibromyalgia	515 390 (7.2)	419 131 (7.1)	59 572 (7.5)	36 687 (8.9)
Hypothyroidism	497 483 (7.0)	433 541 (7.3)	41 042 (5.2)	22 900 (5.5)
Cancer	461 254 (6.5)	402 429 (6.8)	40 223 (5.1)	18 602 (4.5)
Atrial fibrillation	457 273 (6.4)	402 276 (6.8)	38 281 (4.8)	16 716 (4.0)
Anemia	434 419 (6.1)	369 944 (6.2)	43 665 (5.5)	20 810 (5.0)
Substance use disorder	300 196 (4.2)	256 364 (4.3)	26 949 (3.4)	16 883 (4.1)
Deafness and hearing impairment	277 466 (3.9)	253 354 (4.3)	14 920 (1.9)	9192 (2.2)
Migraine	275 381 (3.9)	224 435 (3.8)	24 590 (3.1)	26 356 (6.4)
Heart failure	234 367 (3.3)	194 942 (3.3)	29 301 (3.7)	10 124 (2.4)
Asthma	220 491 (3.1)	187 567 (3.2)	17 837 (2.3)	15 087 (3.6)
Severe mental health disorders	217 139 (3.0)	183 404 (3.1)	19 281 (2.4)	14 454 (3.5)
Liver disease or cirrhosis	175 593 (2.5)	146 723 (2.5)	18 311 (2.3)	10 559 (2.6)
Stroke or transient ischemic attack	127 288 (1.8)	106 773 (1.8)	13 993 (1.8)	6522 (1.6)
Peripheral vascular disease	121 337 (1.7)	106 383 (1.8)	10 892 (1.4)	4062 (1.0)
Dementia	113 746 (1.6)	87 926 (1.5)	17 181 (2.2)	8639 (2.1)
Visual impairments	102 798 (1.4)	90 529 (1.5)	8417 (1.1)	3852 (0.9)
Parkinson disease	64 538 (0.9)	53 102 (0.9)	7601 (1.0)	3835 (0.9)
Epilepsy	54 888 (0.8)	46 328 (0.8)	5294 (0.7)	3266 (0.8)
Viral hepatitis	47 501 (0.7)	40 862 (0.7)	4437 (0.6)	2202 (0.5)
Osteoporosis	50 998 (0.7)	43 432 (0.7)	5146 (0.7)	2420 (0.6)
Pneumonia	26 287 (0.4)	20 439 (0.3)	4536 (0.6)	1312 (0.3)
HIV or AIDS	24 802 (0.3)	18 082 (0.3)	4147 (0.5)	2573 (0.6)
Mobility impairment	24 102 (0.3)	18 247 (0.3)	4147 (0.5)	1708 (0.4)
Pressure and chronic ulcers	20 585 (0.3)	15 808 (0.3)	3385 (0.4)	1392 (0.3)
Acute myocardial infarction	12 683 (0.2)	10 661 (0.2)	1438 (0.2)	584 (0.1)
Multiple sclerosis	17 352 (0.2)	13 466 (0.2)	204 (0.3)	1482 (0.4)
Traumatic brain injury	6006 (0.1)	4873 (0.1)	614 (0.1)	519 (0.1)
Spinal cord injury	2157 (0.03)	1575 (0.03)	438 (0.1)	144 (0.03)

Abbreviations: COPD, chronic obstructive pulmonary disease; PTSD, posttraumatic stress disorder.

^a^ Column totals are presented. Conditions will not sum to 100% within column because multiple conditions can be coded in 1 encounter.

**Figure 1.**
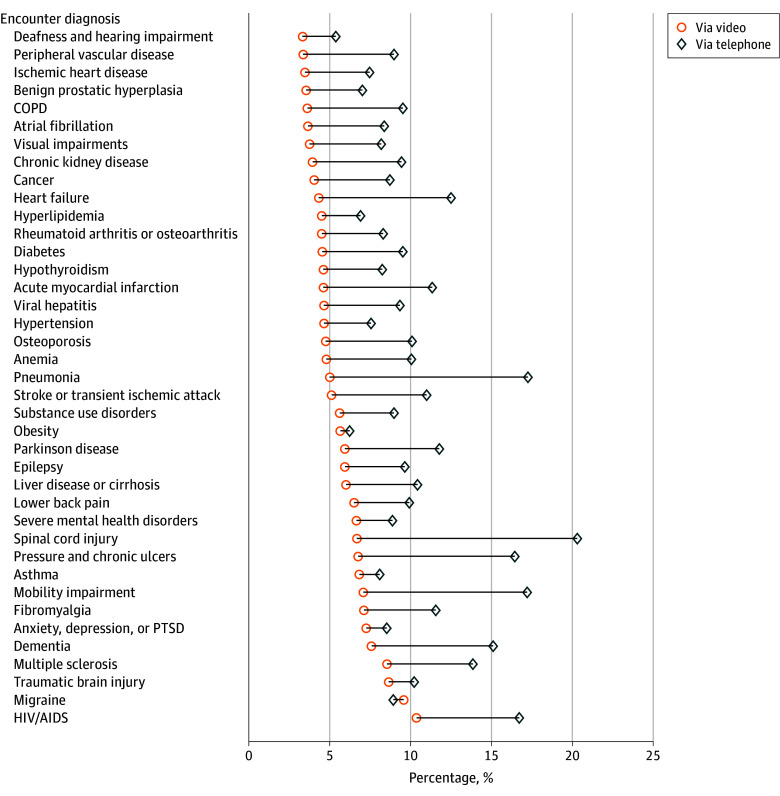
Unadjusted Percentage of Care Completed Via Video and Telephone by Encounter Diagnosis Among 7 144 371 Outpatient Primary Care Encounters Occurring Between April 1, 2022, to March 31, 2023, at the Veterans Health Administration Totals for outpatient encounters by encounter diagnosis can be found in the Table. COPD indicates chronic obstructive pulmonary disease; PTSD, posttraumatic stress disorder.

Conditions with the highest unadjusted percentage of video-based care among the 39 assessed conditions included HIV or AIDs (2573 video encounters of 24 802 encounters [10.4%]), migraine (26 356 of 275 381 encounters [9.6%]), traumatic brain injury (519 of 6006 encounters [8.6%]), and multiple sclerosis (1482 of 17 352 encounters [8.5%]) (Figure 1). Conditions with the lowest percentages of care completed via video included deafness and hearing impairment (9192 of 277 466 encounters [3.3%]), peripheral vascular disease (4062 of 121 337 encounters [3.3%]), and ischemic heart disease (26 798 of 771 811 encounters [3.5%]). Telephone care was more common among all conditions with the exception of migraine encounters, among which 26 356 encounters (9.6%) occurred via video and 24 590 encounters (8.9%) via telephone. Compared with all chronic conditions, high unadjusted percentages of telephone care were present in encounters coded with spinal cord injury (438 of 2157 encounters [20.3%]), pneumonia (4536 of 26 287 encounters [17.3%]), and mobility impairment (4147 of 24 102 encounters [17.2%]) (Figure 1).

**Figure 2.**
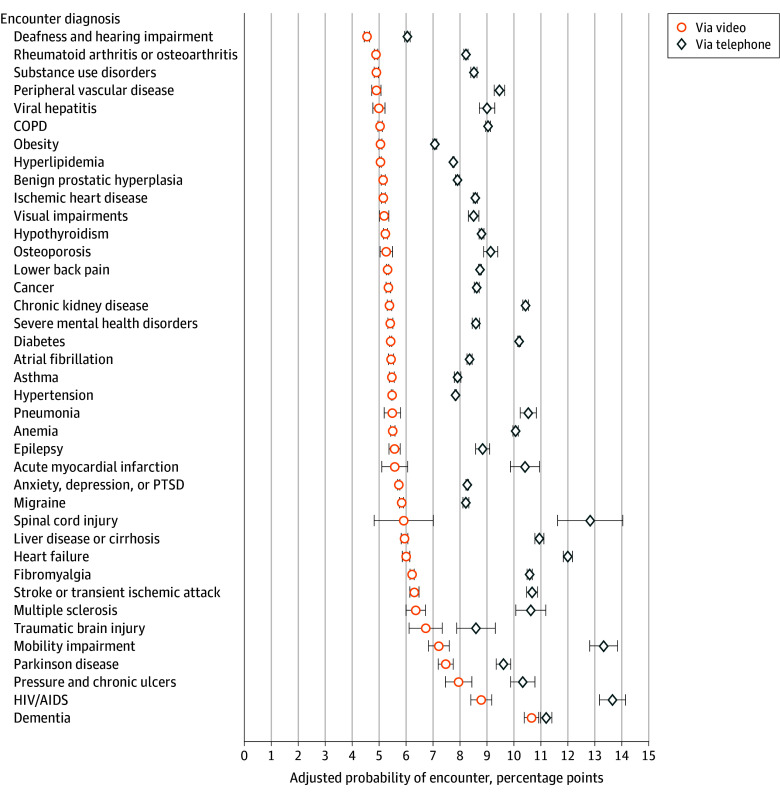
Adjusted Mean Probability of an Encounter Being Telephone- or Video-Based by Encounter Diagnosis Among 7 144 371 Outpatient Primary Care Encounters Occurring Between April 1, 2022, to March 31, 2023, at the Veterans Health Administration (VHA) Separate models were run for video and phone care. All models were adjusted age, gender, race, ethnicity, rurality, marital status, VHA enrollment priority group, drive time from a primary care facility, facility complexity and other chronic conditions managed during the encounter. We also mutually adjusted for the 39 conditions in our model and interpret the estimates for each chronic condition as an independent association. Results are derived from 2 Poisson regressions modeling the likelihood of an encounter being video- or telephone-based when a chronic condition is coded in the encounter diagnoses vs not coded. Estimated margins are reported to illustrate the probability of an encounter occurring via telephone or via video. Adjusted mean probabilities and 95% CIs can be found in eTable 5 and eTable 6 in Supplement 1. COPD indicates chronic obstructive pulmonary disease; PTSD, posttraumatic stress disorder; and error bars, 95% CIs.

**Figure 3.**
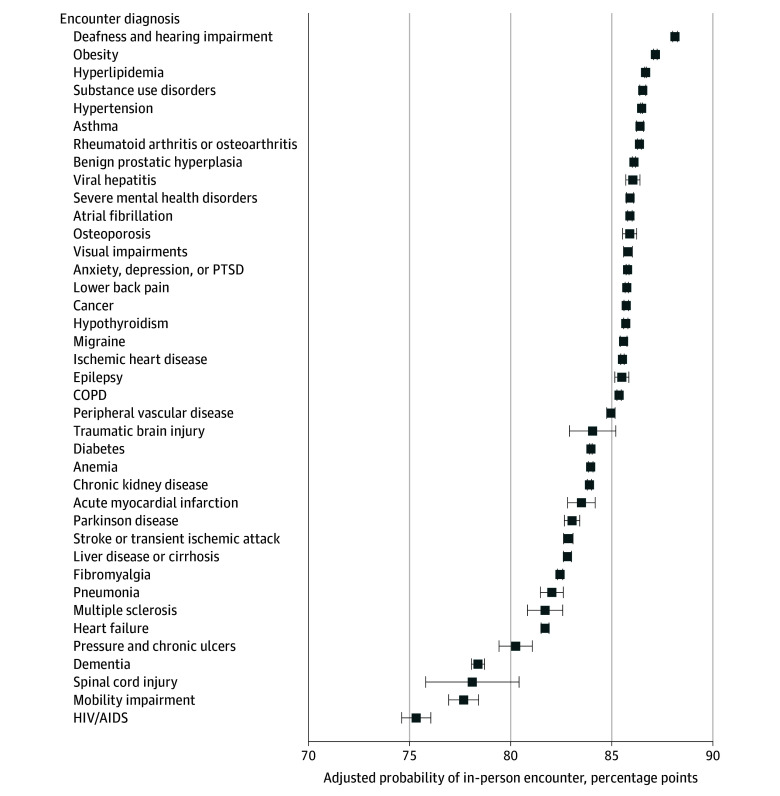
Adjusted Mean Probability of an Encounter Occurring In-Person by Encounter Diagnosis Among 7 144 371 Outpatient Primary Care Encounters Occurring Between April 1, 2022, to March 31, 2023, at the Veterans Health Administration (VHA) Model was adjusted age, gender, race, ethnicity, rurality, marital status, VHA enrollment priority group, drive time from a primary care facility, facility complexity, and other chronic conditions managed during the encounter. Results are derived from a Poisson regression modeling the likelihood of an encounter occurring in-person when a chronic condition is coded in the encounter diagnoses vs not coded. Estimated margins are reported to illustrate the probability of an encounter occurring in-person. Adjusted mean probabilities and 95% CIs can be found in eTable 7 in Supplement 1. COPD, chronic obstructive pulmonary disease; PTSD, posttraumatic stress disorder.

Across all chronic conditions, the adjusted mean probability of an encounter occurring in person was 83.2% (95% CI, 83.1%-84.3%), via telephone was 11.1% (95% CI, 11.0%-11.1%), and via video was 5.8% (95% CI, 5.77%-5.82%). There were differences in the highest adjusted vs unadjusted probability of an encounter being video based when specific conditions were diagnosed. These conditions included dementia (adjusted mean probability, 10.7%; 95% CI, 10.4%-10.9%), HIV or AIDs (adjusted mean probability, 8.8%; 95% CI, 8.4%-9.2%), pressure and chronic ulcers (adjusted mean probability, 8.0%; 95% CI, 7.5%-8.4%), Parkinson disease (adjusted mean probability, 7.5%; 95% CI, 7.2%-7.8%), and mobility impairment (adjusted mean probability, 7.2%; 95% CI, 6.8%-7.6%) (Figure 2). Conditions with the lowest adjusted probability of an encounter being video-based when recorded included deafness and hearing impairment (adjusted mean probability, 4.5%; 95% CI, 4.5%-4.7%), rheumatoid arthritis or osteoarthritis (adjusted mean probability, 4.9%; 95% CI, 4.8%-5.0%), and substance use disorders (adjusted mean probability, 4.9%; 95% CI, 4.8%-5.0%). All 39 conditions had a higher probability of being telephone-based than video-based when each condition was noted in encounter diagnoses (Figure 2). All chronic conditions had at least a 75% probability of being offered in person when the diagnosis was recorded (Figure 3). The top conditions with the highest probability of occurring in person when recorded was similar, but not identical, to those with the lowest amount of video-care, including deafness and hearing impairment (in-person probability: 88.6%; 95% CI, 88.0%-88.2%), obesity (in-person probability: 87.2%; 95% CI, 87.1%-87.3%), and hyperlipidemia (in-person probability: 86.7%; 95% CI, 86.6%-86.7%) (Figure 3).

**Figure 4.**
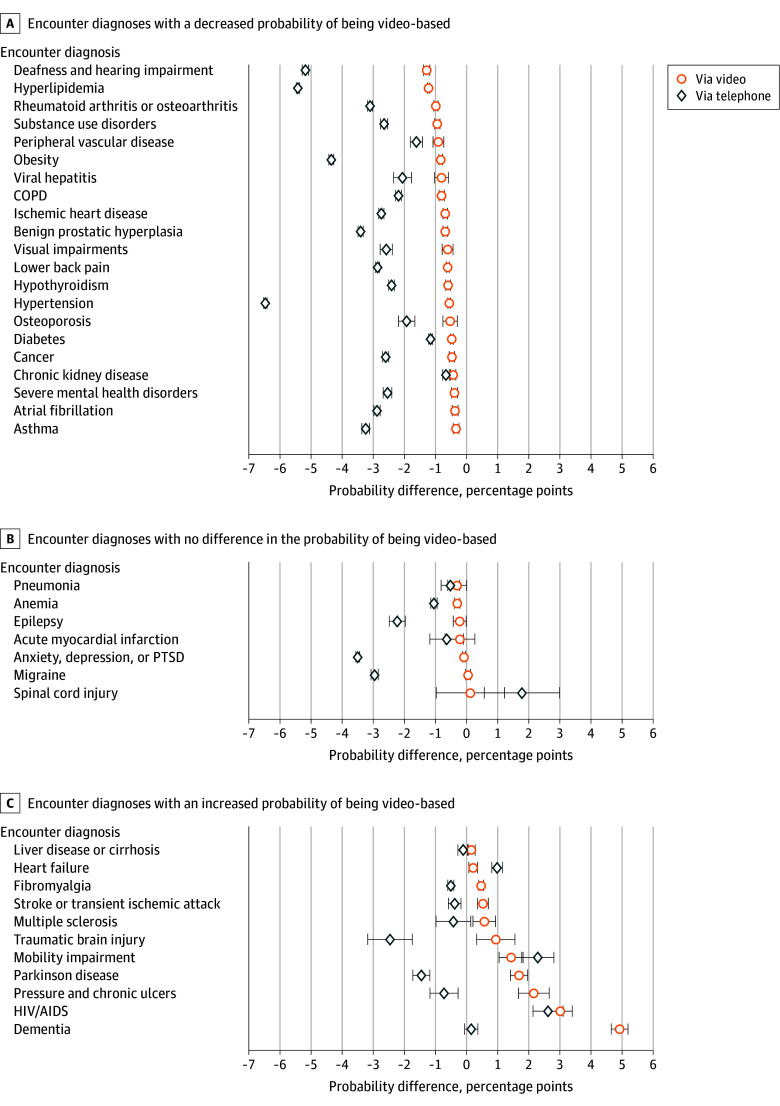
The Difference in the Probability of an Encounter Being Telephone-Based and Video-Based When a Diagnosis Is Coded vs When Not Coded Among 7 144 371 Outpatient Primary Care Encounters Occurring Between April 1, 2022, to March 31, 2023, at the Veterans Health Administration (VHA) Separate models were run for video and phone care. Models were adjusted age, gender, race, ethnicity, rurality, marital status, VHA enrollment priority group, drive time from a primary care facility, facility complexity, and other chronic conditions managed during the encounter. Results are derived from 2 Poisson regressions modeling the likelihood of an encounter being video or telephone-based when a chronic condition is coded in the encounter diagnoses vs not coded. Marginal effects are reported to illustrate the estimated difference in probability of being telephone or video-based. Adjusted mean probabilities and 95% CI can be found in eTable 5 and eTable 6 in Supplement 1. COPD, chronic obstructive pulmonary disease; PTSD, posttraumatic stress disorder; and error bars, 95% CIs.

There were notable variations in the difference of the estimated probability of occurring via telephone or via video when specific diagnoses were coded compared with not coded (Figure 4). A total of 21 conditions had a decreased probability of occurring via telephone and video (Figure 4; eTable 5 and 6 in Supplement 1) and an increased probability of occurring in person (eTable 7 in Supplement 1) when coded in encounter diagnoses. These conditions were often more acute in presentation, required in-person evaluation, or could have serious adverse consequences if not seen early on. These conditions with an increased probability of occurring in person when the diagnosis was noted during an encounter included ischemic heart disease (2.7 [95% CI, 2.6-2.8] percentage points), COPD (2.4 [95% CI, 2.3-2.5] percentage points), atrial fibrillation (2.9 [95% CI, 2.8-3.1] percentage points), cancer (2.7 [95% CI, 2.63-2.86] percentage points), asthma (3.4 [95% CI, 3.2-3.5] percentage points), rheumatoid arthritis or osteoarthritis (3.5 [95% CI, 3.3-3.6] percentage points), and deafness and hearing impairment (5.2 [95% CI, 5.1-5.3] percentage points) (eTable 7 in Supplement 1).

Seven conditions had no difference in the probability of being video-based when the chronic condition was documented in an encounter compared with not documented, including pneumonia; anemia; epilepsy; anxiety, depression, or PTSD; acute myocardial infarction; migraine; and spinal cord injury (Figure 4). The remaining 11 conditions had an increased probability of being a video-based encounter when the condition was documented compared with when the condition was not documented. These included conditions that are often associated with immobility or being home or bed bound: dementia (4.9%; 95% CI, 4.7%-5.2%), pressure and chronic ulcers (2.2% ; 95% CI, 1.67%-2.65%), Parkinson disease (1.7%; 95% CI, 1.41%-1.97%), traumatic brain injury (0.9%; 95% CI, 0.32%-1.55%), and stroke (0.5%; 95% CI, 0.36%-0.70%). Notably, these same conditions had lower probabilities of being telephone-based when noted during an encounter. In contrast, only 3 conditions were associated with a higher probability of being conducted via telehealth (both telephone and video) when documented compared with not documented, including heart failure, HIV or AIDS, and mobility impairment (Figure 4).

## Discussion

In our national assessment of VHA outpatient primary care encounters, we found variation in the probability of a clinical encounter being in person, telephone-based, or video-based, by the clinical conditions documented during that encounter. All conditions had at least a 25% probability of occurring via telehealth, and more telehealth encounters occur via telephone than video. While most assessed chronic conditions were less likely to have been performed via telehealth when a particular diagnosis code was present, many such conditions tended to depend on physical examination evaluations or the need for laboratory assessment. In contrast, clinical conditions associated with physical immobility, and conditions that were less dependent on physical examination, or where transportation to or from a clinical encounter might be a burden had a higher probability of being a video-based interaction than other clinical conditions. These findings suggest that the clinical conditions being addressed during an encounter may be associated with the probability of a health care encounter being in person or via telephone or video.

While patient, facility, and system-level factors have all been shown to impact telehealth use, this evaluation found that specific clinical conditions were associated with the use of telephone and video-based care. We highlighted several important issues in our findings. First, there appeared to be types of conditions that were more likely to be performed in person than via telehealth. Many of these conditions appeared to be those in which a physical examination is often required (eg, lower back pain, COPD, and asthma) or when laboratory or diagnostic evaluations guide therapy (eg, chronic kidney disease, anemia, hypothyroidism). Prior qualitative work among clinicians appeared to validate these findings. For example, Gray et al^9^ reported that patient acuity, the need for labs and testing, and a patient’s health status could be associated with the decision on whether to have a visit in person or via telehealth. Second, there were a number of primary care conditions in which there was a noted increase in the probability of receiving either telephone or video-based care. Many of these conditions included situations where a patient would have physical or functional limitation (eg, mobility impairment, and dementia), thus limiting their ability to attend an in-person clinic appointment. Moreover, conditions, such as Parkinson disease and traumatic brain injury, had a higher probability of video-based care and lower probability of telephone-based care, which suggested that video-based care provided some value for conditions where visual assessment can provide value in the management of that clinical condition.

Of note, there were differences between the unadjusted prevalence and adjusted probability of an encounter occurring via video or telephone when evaluating specific chronic conditions, resulting in some conditions having a higher probability of occurring via video than the unadjusted results would indicate. These differences underscored the role of patient characteristics (eg, age, gender, and rurality of home) and cooccurring chronic conditions as drivers of the likelihood of video or telephone-based care.

These findings have several important clinical implications. As health care systems continue to use and develop more robust telehealth infrastructure, these findings may be helpful in building models, pathways, and guidelines for such programs and clinical care. Currently, few clinical guidelines provide guidance about when to offer telehealth or in-person care,^9,24^ resulting in heterogeneity in the provision of telehealth at the clinician level.^25,26^ These findings, combined with information about patient preferences for the management of specific chronic conditions via telehealth may help guide future work.

Additionally, understanding variations in the use of telehealth is critical to informing future priorities for telehealth reimbursement policies, which remain a major factor of telehealth adoption outside the VHA health care system.^27,28^ Finally, as health care clinicians and policymakers try to find what the right mix of telehealth and in-person care is, the percentage of care delivered via telehealth based on clinical condition may be a valuable measure.^4,26^

### Limitations

This evaluation has several limitations. First, this work was performed in the VHA which cares for an older population that predominantly includes White men, thus decreasing generalizability beyond this setting. Still, the VHA is the nation’s largest single user of telehealth and these findings may guide other health care systems growth of telehealth programs. Second, while we adjusted for characteristics known to be associated with telehealth use, we were unable to account for clinic-level differences, physician-level differences,^29,30^ or patient preferences for telehealth,^31^ which includes coding differences among clinicians or clinics, unobserved patient characteristics, and patient, clinician, or clinic preferences around modality of care that could be associated with our findings. At this time, there is no standard process for selecting the care modality for an encounter. The decision may depend on the availability of appointments, the clinician scheduling the appointment, the clinician preferences for treating a veteran via telehealth, the veteran’s own acceptance of telehealth, and a veteran’s trust in clinicians to treat their condition via telehealth. Third, we present the independent associations for the probability of an encounter being offered via video (or telephone or in person) conditional on all other chronic conditions of interest. While this is a useful way to obtain the contribution of a specific chronic condition, we are unable to understand if combinations of chronic conditions are associated with differential telehealth use. Fourth, *ICD-10* codes recorded during an encounter most likely represent the chronic comorbid burden of a patient, not necessarily the reason for the encounter in primary care. We are unable to understand the extent to which those coded conditions during an encounter were discussed or their severity. Nevertheless, we found noteworthy differences in the probability of an encounter occurring in person, via telephone, or video which suggests that chronic conditions are associated with the likelihood of an encounter occurring in a specific care modality.

## Conclusions

As health care systems continue to refine and optimize their use of telehealth, understanding the impact of clinical diagnosis on telehealth use patterns is important. Our findings suggest that the clinical conditions being addressed during an encounter may impact the probability of a health care encounter being in person, telephone, or video-based.

## Notes

Supplement 1. **eFigure. **Consort flow diagram for study population **eTable 1. **VA managerial cost accounting stop codes used to define outpatient primary care and care modality **eTable 2. **Evaluation and management codes for visits with decision-making by qualified healthcare provider **eTable 3. ***International Classification of Disease Codes* used to classify each encounter’s primary and secondary diagnosis codes **eTable 4. **Frequency of veteran characteristics by 3 975 328 veterans and their corresponding 7 144 371 outpatient primary care encounters occurring between April 1, 2022, to March 31, 2023, at the Veterans Health Administration **eTable 5. **Risk ratios, adjusted mean probability, and the difference in the adjusted mean probability for video-based care by encounter diagnosis among 7 144 371 outpatient primary care encounters at the Veterans Health Administration occurring between April 1, 2022, to March 31, 2023 **eTable 6. **Risk ratios, adjusted mean probability, and the difference in the adjusted mean probability for phone-based care by encounter diagnosis among 7,144,371 outpatient primary care encounters at the Veterans Health Administration occurring between April 1, 2022, to March 31, 2023 **eTable 7. **Risk ratios, adjusted mean probability, and the difference in the adjusted mean probability for in-person care by encounter diagnosis among 7 144 371 outpatient primary care encounters at the Veterans Health Administration occurring between April 1, 2022, to March 31, 2023

Supplement 2.
Data Sharing Statement
