# Supplementary material for: Variability in Primary Care Telehealth Delivery Methods Across Chronic Conditions
Source: JAMA Netw Open. 2025 Mar 26;8(3):e251988. doi: 10.1001/jamanetworkopen.2025.1988 (PMC11947845; doi:10.1001/jamanetworkopen.2025.1988)
Supplement: Supplement 1. — eFigure. Consort flow diagram for study population eTable 1. VA managerial cost accounting stop codes used to define outpatient primary care and care modality eTable 2. Evaluation and management codes for visits with decision-making by qualified healthcare provider eTable 3. International Classification of Disease Codes used to classify each encounter’s primary and secondary diagnosis codes eTable 4. Frequency of veteran characteristics by 3 975 328 veterans and their corresponding 7 144 371 outpatient primary care encounters occurring between April 1, 2022, to March 31, 2023, at the Veterans Health Administration eTable 5. Risk ratios, adjusted mean probability, and the difference in the adjusted mean probability for video-based care by encounter diagnosis among 7 144 371 outpatient primary care encounters at the Veterans Health Administration occurring between April 1, 2022, to March 31, 2023 eTable 6. Risk ratios, adjusted mean probability, and the difference in the adjusted mean probability for phone-based care by encounter diagnosis among 7,144,371 outpatient primary care encounters at the Veterans Health Administration occurring between April 1, 2022, to March 31, 2023 eTable 7. Risk ratios, adjusted mean probability, and the difference in the adjusted mean probability for in-person care by encounter diagnosis among 7 144 371 outpatient primary care encounters at the Veterans Health Administration occurring between April 1, 2022, to March 31, 2023 [file jamanetwopen-e251988-s001.pdf]

Supplemental Online Content

Ferguson JM, Greene L, Van Campen J, Zulman DM, Wray CM. Variability in primary care telehealth delivery methods across chronic conditions. *JAMA Netw Open.* 2025;8(3):e251988. doi:10.1001/jamanetworkopen.2025.1988

eFigure1. Consort flow diagram for study population

eTable 1. VA managerial cost accounting stop codes used to define outpatient primary care and care modality

eTable 2. Evaluation and management codes for visits with decision-making by qualified healthcare provider

eTable 3. *International Classification of Disease Codes* used to classify each encounter's primary and secondary diagnosis codes

eTable 4. Frequency of veteran characteristics by 3 975 328 veterans and their corresponding 7 144 371 outpatient primary care encounters occurring between April 1, 2022, to March 31, 2023 at the Veterans Health Administration

eTable 5. Risk ratios, adjusted mean probability, and the difference in the adjusted mean probability for video-based care by encounter diagnosis among 7 144 371 outpatient primary care encounters at the Veterans Health Administration occurring between April 1, 2022, to March 31, 2023

eTable 6. Risk ratios, adjusted mean probability, and the difference in the adjusted mean probability for phone-based care by encounter diagnosis among 7,144,371 outpatient primary care encounters at the Veterans Health Administration occurring between April 1, 2022, to March 31, 2023

eTable 7. Risk ratios, adjusted mean probability, and the difference in the adjusted mean probability for in-person care by encounter diagnosis among 7 144 371 outpatient primary care encounters at the Veterans Health Administration occurring between April 1, 2022, to March 31, 2023

This supplemental material has been provided by the authors to give readers additional information about their work.

© 2025 Ferguson JM et al. *JAMA Network Open*.

eFigure1: Consort flow diagram for study population in **"Variability in primary care telehealth delivery methods across chronic conditions"**

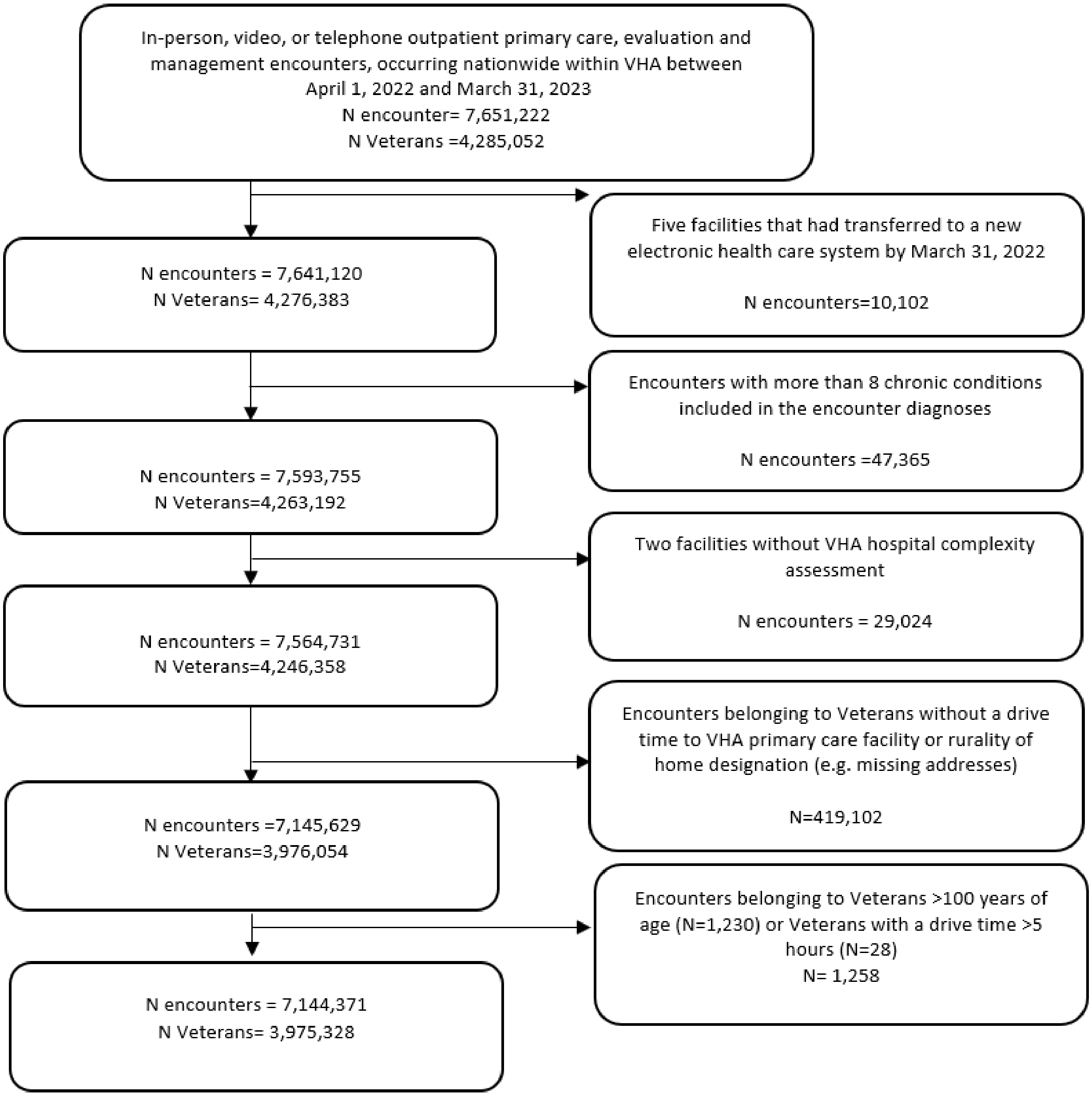

© 2025 Ferguson JM et al. *JAMA Network Open*.

eTable 1: VA Managerial Cost Accounting Stop Codes used to define outpatient primary care and care modality

Codes used to define outpatient primary care

Primary Stop Code IN:<br>170, 171, 172, 178, 318, 322, 323, 338, 348, 350, 704	AND	Secondary Stop Code **NOT** IN:<br>103, 107, 115, 147, 148, 169, 181, 182, 199, 216, 221, 224, 229, 311, 321, 324, 325, 328, 329, 333, 334, 424, 425, 428, 430, 435, 449, 474, 527, 528, 530, 536, 542, 545, 546, 579, 584, 597, 611, 686, 116, 117, 123, 125, 130, 131, 139, 159, 160, 162, 166, 183, 184, 190, 202, 204, 205, 206, 214, 317, 332, 370, 408, 414, 436, 502, 509, 510, 534, 538, 550, 680, 684, 697, 710, 717, 719, 720, 444, 445, 446, 447, 448, 450
**OR**<br>Primary Stop Code:<br>301	AND	Secondary Stop Code IS NULL OR IN:<br>185, 186, 187, 188, 189, 322, 323, 690, 692, 693
**OR**<br>Primary Stop Code:<br>324	AND	Secondary Stop Code IN:<br>301, 309, 322

Codes to define care modality

**Care Delivery Method**	**Stop Codes with Description**
Video	179: Real Time Clinical Video Telehealth To Home - Provider Site,<br>648: Real Time Clinical Video Telehealth With Non-VAMC Location - Provider Site,<br>679: National Center Real Time Clinical Video Telehealth To Home- Provider Sit
Phone	103 Telephone Triage, 147 Telephone/Ancillary, 148 Telephone/Diagnostic, 169 Telephone/Chaplain, 178 Telephone Hbpc, 181 Telephone Dental, 182 Telephone Case Management, 199 Telephone Polytrauma/Traumatic Brain Injury (Tbi), 216 Telephone/Rehabilitation (Rehab) And Support, 221 Telephone Visual Impairment Service Team (Vist), 224 Telephone Spinal Cord Injury (Sci), 229 Telephone/Blind Rehab Program, 324 Telephone/Medicine, 325 Telephone/Neurology, 326 Telephone/Geriatrics, 338 Telephone Primary Care, 424 Telephone/Surgery, 425 Telephone/Prosthetics/Orthotics, 428 Telephone/Optometry, 441 Telephone Anesthesia, 527 Telephone Mental Health, 528 Telephone Homeless Chronically Mentally Ill (Hcmi), 530 Telephone/Hud-VASH, 536 Telephone Mental Health Vocational Assistance, 542 Telephone/Post-Traumatic Stress Disorder (Ptsd), 545 Telephone/Substance Use Disorder, 546 Telephone Intensive Community Mental Health Recovery Services (Icmhr), 579 Telephone/Psychogeriatrics, 584 Telephone Psychosocial Rehabilitation Recovery Center (Prrc), 597 Telephone/Residential Rehabilitation Treatment Program (Rrtp), 611 Telephone/Dialysis, 686 Telephone Contact By Home Telehealth (Ht) Staff, 801 In-VISN, Other VAMC 2nd To 103, 802 Out Of Visn, Va 2ndary To 103
In person	Absence of video or phone stop code†

† and supplementary remote care. Not applicable to this evaluation since we restricted to evaluation and management codes below and specific primary care codes. See Ferguson JM, Jacobs J, Yefimova M, et al. Virtual care expansion in the Veterans Health Administration during the COVID-19 pandemic: clinical services and patient characteristics associated with utilization. *J Am Med Informatics Assoc*. 2021;28(1):453-462. doi:10.1093/jamia/ocaa284 for more details.

© 2025 Ferguson JM et al. *JAMA Network Open*.

eTable 2: Evaluation and management codes for visits with decision-making by qualified healthcare provider

Evaluation and Management CPT (Current Procedural Terminology) codes used include:	99201, 99202, 99203, 99204, 99205, 99211, 99212, 99213, 99214, 99215, 99421, 99422, 99423, 99441, 99442, 99443.

© 2025 Ferguson JM et al. *JAMA Network Open*.

eTable 3: International Classification of Disease Codes Used to classify each encounter's primary and secondary diagnosis codes

We chose 39 conditions that represented the most common chronic conditions among Veterans.

Definitions were derived from the Center for Medicare and Medicaid Services (CMS) Chronic Condition Warehouse (CCW)[13] and from H-CUP Clinical Classification Software Refined (CCSR).
CCW has two lists of chronic conditions at Condition Categories - Chronic Conditions Data Warehouse (ccwdata.org). Lower Back Pain was included given its high prevalence among Veterans and its definition was sourced from H-CUP's CCSR diagnosis categories Clinical Classifications Software Refined (CCSR) for ICD-10-CM Diagnoses (ahrq.gov).

All the CCW cancer conditions and hepatitis were combined into a single condition. Several of the CCW MH conditions were combined for this study. Other conditions from CCW were not included in our chronic conditions assessed given their low prevalence among Veterans in primary care settings such as Learning disability, hip fractures, sickle cell disease, and COVID-19 (represents acute infection rather than a chronic disease/long-COVID).

**Condition (ABBR)***	**ICD10Codes**
Acute Myocardial Infarction (AMI)	I21.01, I21.02, I21.09, I21.11, I21.19, I21.21, I21.29, I21.3, I21.4, I21.9, I21.A1, I21.A9, I22.0, I22.1, I22.2, I22.8, I22.9, I23.0, I23.1, I23.2, I23.3, I23.4, I23.5, I23.6, I23.7, I23.8
Anemia (ANEMIA)	C94.6, D46.0, D46.1, D46.20, D46.21, D46.22, D46.4, D46.9, D46.A, D46.B, D46.C, D46.Z, D47.4, D50.0, D50.1, D50.8, D50.9, D51.0, D51.1, D51.2, D51.3, D51.8, D51.9, D52.0, D52.1, D52.8, D52.9, D53.0, D53.1, D53.2, D53.8, D53.9, D55.0, D55.1, D55.2, D55.21, D55.29, D55.3, D55.8, D55.9, D56.0, D56.1, D56.2, D56.3, D56.4, D56.5, D56.8, D56.9, D57.00, D57.01, D57.02, D57.1, D57.20, D57.211, D57.212, D57.219, D57.3, D57.40, D57.411, D57.412, D57.413, D57.418, D57.419, D57.42, D57.431, D57.432, D57.433, D57.438, D57.439, D57.44, D57.451, D57.452, D57.453, D57.458, D57.459, D57.80, D57.811, D57.812, D57.813, D57.818, D57.819, D58.0, D58.1, D58.2, D58.8, D58.9, D59.0, D59.1, D59.10, D59.11, D59.12, D59.13, D59.19, D59.2, D59.3, D59.4, D59.5, D59.6, D59.8, D59.9, D60.0, D60.1, D60.8, D60.9, D61.01, D61.09, D61.1, D61.2, D61.3, D61.810, D61.811, D61.818, D61.82, D61.89, D61.9, D63.0, D63.1, D63.8, D64.0, D64.1, D64.2, D64.3, D64.4, D64.81, D64.89, D64.9, D75.81
Combo of ANXIETY, DEPRESS, and PTSD (ANXDEPPTSD)	F06.4, F40.00, F40.01, F40.02, F40.10, F40.11, F40.210, F40.218, F40.220, F40.228, F40.230, F40.231, F40.232, F40.233, F40.240, F40.241, F40.242, F40.243, F40.248, F40.290, F40.291, F40.298, F40.8, F40.9, F41.0, F41.1, F41.3, F41.8, F41.9, F42., F42.2, F42.3, F42.4, F42.8, F42.9, F43.0, F44.9, F45.8, F48.8, F48.9, F93.8, F99., R45.2, R45.5, R45.6, R45.7, F06.31, F06.32, F32.0, F32.1, F32.2, F32.3, F32.4, F32.5, F32.8, F32.89, F32.9, F32.A, F33.0, F33.1, F33.2, F33.3, F33.40, F33.41, F33.42, F33.9, F34.0, F34.1, F43.21, F43.23, F43.10, F43.11, F43.12
Asthma (ASTHMA)	J45.20, J45.21, J45.22, J45.30, J45.31, J45.32, J45.40, J45.41, J45.42, J45.50, J45.51, J45.52, J45.901, J45.902, J45.909, J45.990, J45.991, J45.998
Atrial Fibrillation and Flutter (ATRIAL_FIB)	I48.0, I48.1, I48.11, I48.19, I48.2, I48.20, I48.21, I48.3, I48.4, I48.91
Benign Prostatic Hyperplasia (BPH)	N40.0, N40.1, N40.2, N40.3

© 2025 Ferguson JM et al. *JAMA Network Open*.

Traumatic Brain Injury and Nonpsychotic Mental Disorders due to Brain Damage (BRAINJ)	F07.0, F07.81, F07.89, F48.2, S04.011S, S04.012S, S04.019S, S04.02XS, S04.031S, S04.032S, S04.039S, S04.041S, S04.042S, S04.049S, S04.10XS, S04.11XS, S04.12XS, S04.20XS, S04.21XS, S04.22XS, S04.30XS, S04.31XS, S04.32XS, S04.40XS, S04.41XS, S04.42XS, S04.50XS, S04.51XS, S04.52XS, S04.60XS, S04.61XS, S04.62XS, S04.70XS, S04.71XS, S04.72XS, S04.811S, S04.812S, S04.819S, S04.891S, S04.892S, S04.899S, S04.9XXS, S06.0X0S, S06.0X1S, S06.0X2S, S06.0X3S, S06.0X4S, S06.0X5S, S06.0X6S, S06.0X7S, S06.0X8S, S06.0X9S, S06.1X0S, S06.1X1S, S06.1X2S, S06.1X3S, S06.1X4S, S06.1X5S, S06.1X6S, S06.1X7S, S06.1X8S, S06.1X9S, S06.2X0S, S06.2X1S, S06.2X2S, S06.2X3S, S06.2X4S, S06.2X5S, S06.2X6S, S06.2X7S, S06.2X8S, S06.2X9S, S06.300S, S06.301S, S06.302S, S06.303S, S06.304S, S06.305S, S06.306S, S06.307S, S06.308S, S06.309S, S06.310S, S06.311S, S06.312S, S06.313S, S06.314S, S06.315S, S06.316S, S06.317S, S06.318S, S06.319S, S06.320S, S06.321S, S06.322S, S06.323S, S06.324S, S06.325S, S06.326S, S06.327S, S06.328S, S06.329S, S06.330S, S06.331S, S06.332S, S06.333S, S06.334S, S06.335S, S06.336S, S06.337S, S06.338S, S06.339S, S06.340S, S06.341S, S06.342S, S06.343S, S06.344S, S06.345S, S06.346S, S06.347S, S06.348S, S06.349S, S06.350S, S06.351S, S06.352S, S06.353S, S06.354S, S06.355S, S06.356S, S06.357S, S06.358S, S06.359S, S06.360S, S06.361S, S06.362S, S06.363S, S06.364S, S06.365S, S06.366S, S06.367S, S06.368S, S06.369S, S06.370S, S06.371S, S06.372S, S06.373S, S06.374S, S06.375S, S06.376S, S06.377S, S06.378S, S06.379S, S06.380S, S06.381S, S06.382S, S06.383S, S06.384S, S06.385S, S06.386S, S06.387S, S06.388S, S06.389S, S06.4X0S, S06.4X1S, S06.4X2S, S06.4X3S, S06.4X4S, S06.4X5S, S06.4X6S, S06.4X7S, S06.4X8S, S06.4X9S, S06.5X0S, S06.5X1S, S06.5X2S, S06.5X3S, S06.5X4S, S06.5X5S, S06.5X6S, S06.5X7S, S06.5X8S, S06.5X9S, S06.6X0S, S06.6X1S, S06.6X2S, S06.6X3S, S06.6X4S, S06.6X5S, S06.6X6S, S06.6X7S, S06.6X8S, S06.6X9S, S06.810S, S06.811S, S06.812S, S06.813S, S06.814S, S06.815S, S06.816S, S06.817S, S06.818S, S06.819S, S06.820S, S06.821S, S06.822S, S06.823S
Cancer (CANCER)	C18.0, C18.1, C18.2, C18.3, C18.4, C18.5, C18.6, C18.7, C18.8, C18.9, C19., C20., C34.00, C34.01, C34.02, C34.10, C34.11, C34.12, C34.2, C34.30, C34.31, C34.32, C34.80, C34.81, C34.82, C34.90, C34.91, C34.92, C49.A4, C49.A5, C50.011, C50.012, C50.019, C50.021, C50.022, C50.029, C50.111, C50.112, C50.119, C50.121, C50.122, C50.129, C50.211, C50.212, C50.219, C50.221, C50.222, C50.229, C50.311, C50.312, C50.319, C50.321, C50.322, C50.329, C50.411, C50.412, C50.419, C50.421, C50.422, C50.429, C50.511, C50.512, C50.519, C50.521, C50.522, C50.529, C50.611, C50.612, C50.619, C50.621, C50.622, C50.629, C50.811, C50.812, C50.819, C50.821, C50.822, C50.829, C50.911, C50.912, C50.919, C50.921, C50.922, C50.929, C54.0, C54.1, C54.2, C54.3, C54.8, C54.9, C61., C64.1, C64.2, C64.9, C65.1, C65.2, C65.9, C66.1, C66.2, C66.9, C68.8, C68.9, C81.00, C81.01, C81.02, C81.03, C81.04, C81.05, C81.06, C81.07, C81.08, C81.09, C81.10, C81.11, C81.12, C81.13, C81.14, C81.15, C81.16, C81.17, C81.18, C81.19, C81.20, C81.21, C81.22, C81.23, C81.24, C81.25, C81.26, C81.27, C81.28, C81.29, C81.30, C81.31, C81.32, C81.33, C81.34, C81.35, C81.36, C81.37, C81.38, C81.39, C81.40, C81.41, C81.42, C81.43, C81.44, C81.45, C81.46, C81.47, C81.48, C81.49, C81.70, C81.71, C81.72, C81.73, C81.74, C81.75, C81.76, C81.77, C81.78, C81.79, C81.90, C81.91, C81.92, C81.93, C81.94, C81.95, C81.96, C81.97, C81.98, C81.99, C82.00, C82.01, C82.02, C82.03, C82.04, C82.05, C82.06, C82.07, C82.08, C82.09, C82.10, C82.11, C82.12, C82.13, C82.14, C82.15, C82.16, C82.17, C82.18, C82.19, C82.20, C82.21, C82.22, C82.23, C82.24, C82.25, C82.26, C82.27, C82.28, C82.29, C82.30, C82.31, C82.32, C82.33, C82.34, C82.35, C82.36, C82.37, C82.38, C82.39, C82.40, C82.41, C82.42, C82.43, C82.44, C82.45, C82.46, C82.47, C82.48, C82.49, C82.50, C82.51, C82.52, C82.53, C82.54, C82.55, C82.56, C82.57, C82.58, C82.59, C82.60, C82.61,

© 2025 Ferguson JM et al. *JAMA Network Open*.

	C82.62, C82.63, C82.64, C82.65, C82.66, C82.67, C82.68, C82.69, C82.80, C82.81, C82.82, C82.83, C82.84, C82.85, C82.86, C82.87, C82.88, C82.89, C82.90, C82.
Chronic Kidney Disease (CHRONICKIDNEY)	A18.11, A52.75, B52.0, E08.21, E08.22, E08.29, E09.21, E09.22, E09.29, E10.21, E10.22, E10.29, E11.21, E11.22, E11.29, E13.21, E13.22, E13.29, I12.0, I12.9, I13.0, I13.10, I13.11, I13.2, K76.7, M10.30, M10.311, M10.312, M10.319, M10.321, M10.322, M10.329, M10.331, M10.332, M10.339, M10.341, M10.342, M10.349, M10.351, M10.352, M10.359, M10.361, M10.362, M10.369, M10.371, M10.372, M10.379, M10.38, M10.39, M32.14, M32.15, M35.04, M35.0A, N01.0, N01.1, N01.2, N01.3, N01.4, N01.5, N01.6, N01.7, N01.8, N01.9, N01.A, N02.0, N02.1, N02.2, N02.3, N02.4, N02.5, N02.6, N02.7, N02.8, N02.9, N02.A, N03.0, N03.1, N03.2, N03.3, N03.4, N03.5, N03.6, N03.7, N03.8, N03.9, N03.A, N04.0, N04.1, N04.2, N04.3, N04.4, N04.5, N04.6, N04.7, N04.8, N04.9, N04.A, N05.0, N05.1, N05.2, N05.3, N05.4, N05.5, N05.6, N05.7, N05.8, N05.9, N05.A, N06.0, N06.1, N06.2, N06.3, N06.4, N06.5, N06.6, N06.7, N06.8, N06.9, N06.A, N07.0, N07.1, N07.2, N07.3, N07.4, N07.5, N07.6, N07.7, N07.8, N07.9, N07.A, N08., N14.0, N14.1, N14.2, N14.3, N14.4, N15.0, N15.8, N15.9, N16., N18.1, N18.2, N18.3, N18.30, N18.31, N18.32, N18.4, N18.5, N18.6, N18.9, N25.1, N25.89, N25.9, N26.1, N26.9, N99.0, Q61.02, Q61.11, Q61.19, Q61.2, Q61.3, Q61.4, Q61.5, Q61.8
Chronic Obstructive Pulmonary Disease (COPD)	J40., J41.0, J41.1, J41.8, J42., J43.0, J43.1, J43.2, J43.9, J44.0, J44.1, J44.9, J47.0, J47.1, J47.9, J98.2, J98.3
Dementia, including Alzheimers (DEMENTIA)	F01.50, F01.51, F02.80, F02.81, F03.90, F03.91, F05., G13.8, G30.0, G30.1, G30.8, G30.9, G31.01, G31.09, G31.1, G31.2, G31.83, G94., R41.81
Diabetes (DIABETES)	E08.00, E08.01, E08.10, E08.11, E08.21, E08.22, E08.29, E08.311, E08.319, E08.321, E08.3211, E08.3212, E08.3213, E08.3219, E08.329, E08.3291, E08.3292, E08.3293, E08.3299, E08.331, E08.3311, E08.3312, E08.3313, E08.3319, E08.339, E08.3391, E08.3392, E08.3393, E08.3399, E08.341, E08.3411, E08.3412, E08.3413, E08.3419, E08.349, E08.3491, E08.3492, E08.3493, E08.3499, E08.351, E08.3511, E08.3512, E08.3513, E08.3519, E08.3521, E08.3522, E08.3523, E08.3529, E08.3531, E08.3532, E08.3533, E08.3539, E08.3541, E08.3542, E08.3543, E08.3549, E08.3551, E08.3552, E08.3553, E08.3559, E08.359, E08.3591, E08.3592, E08.3593, E08.3599, E08.36, E08.37X1, E08.37X2, E08.37X3, E08.37X9, E08.39, E08.40, E08.41, E08.42, E08.43, E08.44, E08.49, E08.51, E08.52, E08.59, E08.610, E08.618, E08.620, E08.621, E08.622, E08.628, E08.630, E08.638, E08.641, E08.649, E08.65, E08.69, E08.8, E08.9, E09.00, E09.01, E09.10, E09.11, E09.21, E09.22, E09.29, E09.311, E09.319, E09.321, E09.3211, E09.3212, E09.3213, E09.3219, E09.329, E09.3291, E09.3292, E09.3293, E09.3299, E09.331, E09.3311, E09.3312, E09.3313, E09.3319, E09.339, E09.3391, E09.3392, E09.3393, E09.3399, E09.341, E09.3411, E09.3412, E09.3413, E09.3419, E09.349, E09.3491, E09.3492, E09.3493, E09.3499, E09.351, E09.3511, E09.3512, E09.3513, E09.3519, E09.3521, E09.3522, E09.3523, E09.3529, E09.3531, E09.3532,

© 2025 Ferguson JM et al. *JAMA Network Open*.

	E09.3533, E09.3539, E09.3541, E09.3542, E09.3543, E09.3549, E09.3551, E09.3552, E09.3553, E09.3559, E09.359, E09.3591, E09.3592, E09.3593, E09.3599, E09.36, E09.37X1, E09.37X2, E09.37X3, E09.37X9, E09.39, E09.40, E09.41, E09.42, E09.43, E09.44, E09.49, E09.51, E09.52, E09.59, E09.610, E09.618, E09.620, E09.621, E09.622, E09.628, E09.630, E09.638, E09.641, E09.649, E09.65, E09.69, E09.8, E09.9, E10.10, E10.11, E10.21, E10.22, E10.29, E10.311, E10.319, E10.321, E10.3211, E10.3212, E10.3213, E10.3219, E10.329, E10.3291, E10.3292, E10.3293, E10.3299, E10.331, E10.3311, E10.3312, E10.3313, E10.3319, E10.339, E10.3391, E10.3392, E10.3393, E10.3399, E10.341, E10.3411, E10.3412, E10.3413, E10.341
Epilepsy (EPILEP)	G40.001, G40.009, G40.011, G40.019, G40.101, G40.109, G40.111, G40.119, G40.201, G40.209, G40.211, G40.219, G40.301, G40.309, G40.311, G40.319, G40.401, G40.409, G40.411, G40.419, G40.42, G40.501, G40.509, G40.801, G40.802, G40.803, G40.804, G40.811, G40.812, G40.813, G40.814, G40.821, G40.822, G40.823, G40.824, G40.833, G40.834, G40.89, G40.901, G40.909, G40.911, G40.919, G40.A01, G40.A09, G40.A11, G40.A19, G40.B01, G40.B09, G40.B11, G40.B19
Fibromyalgia and Chronic Pain and Fatigue (FIBRO)	G89.21, G89.22, G89.28, G89.29, G89.3, G89.4, M54.10, M54.11, M54.12, M54.13, M54.14, M54.15, M54.16, M54.17, M54.18, M60.80, M60.811, M60.812, M60.819, M60.821, M60.822, M60.829, M60.831, M60.832, M60.839, M60.841, M60.842, M60.849, M60.851, M60.852, M60.859, M60.861, M60.862, M60.869, M60.871, M60.872, M60.879, M60.88, M60.89, M60.9, M79.1, M79.10, M79.11, M79.12, M79.18, M79.2, M79.7, R53.82
Sensory — Deafness and Hearing Impairment (HEARIM)	H90.3, H90.41, H90.42, H90.5, H90.6, H90.71, H90.72, H90.8, H90.A21, H90.A22, H90.A31, H90.A32, H91.01, H91.02, H91.03, H91.09, H91.3, H91.8X1, H91.8X2, H91.8X3, H91.8X9, H91.90, H91.91, H91.92, H91.93
Viral Hepatitis (General), including: (HEPVIRAL)	B15.0, B15.9, B16.0, B16.1, B16.2, B16.9, B17.0, B17.10, B17.11, B17.2, B17.8, B17.9, B18.0, B18.1, B18.2, B18.8, B18.9, B19.0, B19.10, B19.11, B19.20, B19.21, B19.9, Z22.50, Z22.51, Z22.52, Z22.59
Heart Failure and Non-Ischemic Heart Disease (HF)	I09.81, I11.0, I13.0, I13.2, I42.0, I42.5, I42.6, I42.7, I42.8, I43., I50.1, I50.20, I50.21, I50.22, I50.23, I50.30, I50.31, I50.32, I50.33, I50.40, I50.41, I50.42, I50.43, I50.810, I50.811, I50.812, I50.813, I50.814, I50.82, I50.83, I50.84, I50.89, I50.9, P29.0
Human Immunodeficiency Virus and/or Acquired Immunodeficiency Syndrome (HIV/AIDS) (HIVAIDS)	B20., B97.35, R75., Z21.
Hyperlipidemia (HLP)	E78.0, E78.00, E78.01, E78.1, E78.2, E78.3, E78.4, E78.41, E78.49, E78.5
Hypertension (HTN)	H35.031, H35.032, H35.033, H35.039, I10., I11.0, I11.9, I12.0, I12.9, I13.0, I13.10, I13.11, I13.2, I15.0, I15.1, I15.2, I15.8, I15.9, I67.4, N26.2
Hypothyroidism (HYPTHYRD)	E00.0, E00.1, E00.2, E00.9, E01.8, E02., E03.0, E03.1, E03.2, E03.3, E03.4, E03.8, E03.9, E89.0
Ischemic Heart Disease (ISCHEMICHEART)	I20.0, I20.1, I20.8, I24.0, I24.1, I24.8, I25.10, I25.110, I25.111, I25.118, I25.119, I25.3, I25.41, I25.42, I25.5, I25.6, I25.700, I25.701, I25.708, I25.710, I25.711, I25.718, I25.719, I25.720, I25.721, I25.728, I25.729, I25.730, I25.731, I25.738, I25.739, I25.750, I25.751, I25.758, I25.759, I25.760, I25.761, I25.768, I25.769, I25.790, I25.791, I25.798, I25.799, I25.810, I25.811, I25.812, I25.82, I25.83, I25.84, I25.89, I25.9
Lower back pain (LBP)**	M51.06, M51.16, M51.17, M51.26, M51.27, M51.36, M51.37, M51.46, M51.47, M51.86, M51.87, M53.2X6, M53.2X7, M53.2X8, M53.3, M53.86, M53.87, M53.88, M54.16, M54.17, M54.18, M54.30, M54.31, M54.32, M54.40, M54.41, M54.42, M54.5, M54.50, M54.51, M54.59, M62.830

© 2025 Ferguson JM et al. *JAMA Network Open*.

Liver Disease, Cirrhosis, and Other Liver Conditions (except Viral Hepatitis) (LIVER)	K70.0, K70.10, K70.11, K70.2, K70.30, K70.31, K70.40, K70.41, K70.9, K71.0, K71.11, K71.7, K71.8, K71.9, K72.00, K72.01, K72.10, K72.11, K72.90, K72.91, K74.0, K74.00, K74.01, K74.02, K74.1, K74.2, K74.3, K74.4, K74.5, K74.60, K74.69, K75.0, K75.1, K75.81, K75.89, K75.9, K76.0, K76.1, K76.2, K76.3, K76.5, K76.6, K76.7, K76.81, K76.89, K76.9, K77., K80.30, K80.31, K80.32, K80.33, K80.34, K80.35, K80.36, K80.37, K83.0, R16.0, R16.2, Z48.23, Z94.4
Migraine and Chronic Headache (MIGRAINE)	G43.001, G43.009, G43.011, G43.019, G43.101, G43.109, G43.111, G43.119, G43.401, G43.409, G43.411, G43.419, G43.501, G43.509, G43.511, G43.519, G43.601, G43.609, G43.611, G43.619, G43.701, G43.709, G43.711, G43.719, G43.801, G43.809, G43.811, G43.819, G43.821, G43.829, G43.831, G43.839, G43.901, G43.909, G43.911, G43.919, G43.A0, G43.A1, G43.B0, G43.B1, G43.C0, G43.C1, G43.D0, G43.D1, G44.001, G44.009, G44.011, G44.019, G44.021, G44.029, G44.031, G44.039, G44.041, G44.049, G44.051, G44.059, G44.091, G44.099, G44.1, G44.201, G44.209, G44.211, G44.219, G44.221, G44.229, G44.301, G44.309, G44.311, G44.319, G44.321, G44.329, G44.40, G44.41, G44.51, G44.52, G44.53, G44.59, G44.81, G44.82, G44.83, G44.84, G44.85, G44.86, G44.89
Mobility Impairments (MOBIMP)	G04.1, G11.4, G81.00, G81.01, G81.02, G81.03, G81.04, G81.10, G81.11, G81.12, G81.13, G81.14, G81.90, G81.91, G81.92, G81.93, G81.94, G82.20, G82.21, G82.22, G82.50, G82.51, G82.52, G82.53, G82.54, G83.0, G83.10, G83.11, G83.12, G83.13, G83.14, G83.20, G83.21, G83.22, G83.23, G83.24, G83.30, G83.31, G83.32, G83.33, G83.34, G83.4, G83.5, G83.81, G83.82, G83.83, G83.84, G83.89, G83.9, I69.031, I69.032, I69.033, I69.034, I69.039, I69.041, I69.042, I69.043, I69.044, I69.049, I69.051, I69.052, I69.053, I69.054, I69.059, I69.061, I69.062, I69.063, I69.064, I69.065, I69.069, I69.131, I69.132, I69.133, I69.134, I69.139, I69.141, I69.142, I69.143, I69.144, I69.149, I69.151, I69.152, I69.153, I69.154, I69.159, I69.161, I69.162, I69.163, I69.164, I69.165, I69.169, I69.231, I69.232, I69.233, I69.234, I69.239, I69.241, I69.242, I69.243, I69.244, I69.249, I69.251, I69.252, I69.253, I69.254, I69.259, I69.261, I69.262, I69.263, I69.264, I69.265, I69.269, I69.331, I69.332, I69.333, I69.334, I69.339, I69.341, I69.342, I69.343, I69.344, I69.349, I69.351, I69.352, I69.353, I69.354, I69.359, I69.361, I69.362, I69.363, I69.364, I69.365, I69.369, I69.831, I69.832, I69.833, I69.834, I69.839, I69.841, I69.842, I69.843, I69.844, I69.849, I69.851, I69.852, I69.853, I69.854, I69.859, I69.861, I69.862, I69.863, I69.864, I69.865, I69.869, I69.931, I69.932, I69.933, I69.934, I69.939, I69.941, I69.942, I69.943, I69.944, I69.949, I69.951, I69.952, I69.953, I69.954, I69.959, I69.961, I69.962, I69.963, I69.964, I69.965, I69.969
Multiple Sclerosis and Transverse Myelitis (MULSCL)	G35., G36.0, G36.1, G36.8, G36.9, G37.1, G37.2, G37.3, G37.4, G37.8, G37.9
Obesity (OBESITY)	E66.01, E66.09, E66.1, E66.2, E66.8, E66.9, Z68.30, Z68.31, Z68.32, Z68.33, Z68.34, Z68.35, Z68.36, Z68.37, Z68.38, Z68.39, Z68.41, Z68.42, Z68.43, Z68.44, Z68.45
Osteoporosis With or Without Pathological Fracture (OSTEOPOROSIS)	M80.00XA, M80.011A, M80.012A, M80.019A, M80.021A, M80.022A, M80.029A, M80.031A, M80.032A, M80.039A, M80.041A, M80.042A, M80.049A, M80.051A, M80.052A, M80.059A, M80.061A, M80.062A, M80.069A, M80.071A, M80.072A, M80.079A, M80.08XA, M80.0AXA, M80.80XA, M80.811A, M80.812A, M80.819A, M80.821A, M80.822A, M80.829A, M80.831A, M80.832A, M80.839A, M80.841A, M80.842A, M80.849A, M80.851A, M80.852A, M80.859A, M80.861A, M80.862A, M80.869A, M80.871A, M80.872A, M80.879A, M80.88XA, M80.8AXA, M81.0, M81.6, M81.8

© 2025 Ferguson JM et al. *JAMA Network Open*.

Pneumonia, All-cause (PNEUMO)	A01.03, A02.22, A06.5, A20.2, A21.2, A22.1, A31.0, A37.01, A37.11, A37.81, A37.91, A40.3, A42.0, A43.0, A48.1, A50.04, A54.84, B01.2, B05.2, B06.81, B37.1, B38.0, B38.2, B39.0, B39.2, B40.0, B40.2, B41.0, B58.3, B59., B66.4, B67.1, B77.81, B95.3, B96.0, B96.1, J09.X1, J10.00, J10.01, J10.08, J11.00, J11.08, J12.0, J12.1, J12.2, J12.3, J12.81, J12.82, J12.89, J12.9, J13., J14., J15.0, J15.1, J15.20, J15.211, J15.212, J15.29, J15.3, J15.4, J15.5, J15.6, J15.7, J15.8, J15.9, J16.0, J16.8, J17., J18.0, J18.1, J18.2, J18.8, J18.9, J20.0, J84.111, J84.116, J84.117, J84.178, J84.2, J85.1, J95.851, P23.0, P23.1, P23.2, P23.3, P23.4, P23.5, P23.6, P23.8, P23.9, Z87.01
Parkinson's Disease and Secondary Parkinsonism (PRKNSN)	G20., G21.11, G21.19, G21.3, G21.4, G21.8, G21.9, G31.83
Peripheral Vascular Disease (PVD) (PVD)	E08.51, E08.52, E09.51, E09.52, E10.51, E10.52, E11.51, E11.52, E13.51, E13.52, I70.0, I70.1, I70.201, I70.202, I70.203, I70.208, I70.209, I70.211, I70.212, I70.213, I70.218, I70.219, I70.221, I70.222, I70.223, I70.228, I70.229, I70.231, I70.232, I70.233, I70.234, I70.235, I70.238, I70.239, I70.241, I70.242, I70.243, I70.244, I70.245, I70.248, I70.249, I70.25, I70.291, I70.292, I70.293, I70.298, I70.299, I70.92, I73.81, I73.89, I73.9, I79.1, I79.8
Rheumatoid Arthritis/Osteoarthritis (RA_OA)	L40.50, L40.51, L40.54, L40.59, M05.00, M05.011, M05.012, M05.019, M05.021, M05.022, M05.029, M05.031, M05.032, M05.039, M05.041, M05.042, M05.049, M05.051, M05.052, M05.059, M05.061, M05.062, M05.069, M05.071, M05.072, M05.079, M05.09, M05.10, M05.111, M05.112, M05.119, M05.121, M05.122, M05.129, M05.131, M05.132, M05.139, M05.141, M05.142, M05.149, M05.151, M05.152, M05.159, M05.161, M05.162, M05.169, M05.171, M05.172, M05.179, M05.19, M05.20, M05.211, M05.212, M05.219, M05.221, M05.222, M05.229, M05.231, M05.232, M05.239, M05.241, M05.242, M05.249, M05.251, M05.252, M05.259, M05.261, M05.262, M05.269, M05.271, M05.272, M05.279, M05.29, M05.30, M05.311, M05.312, M05.319, M05.321, M05.322, M05.329, M05.331, M05.332, M05.339, M05.341, M05.342, M05.349, M05.351, M05.352, M05.359, M05.361, M05.362, M05.369, M05.371, M05.372, M05.379, M05.39, M05.40, M05.411, M05.412, M05.419, M05.421, M05.422, M05.429, M05.431, M05.432, M05.439, M05.441, M05.442, M05.449, M05.451, M05.452, M05.459, M05.461, M05.462, M05.469, M05.471, M05.472, M05.479, M05.49, M05.50, M05.511, M05.512, M05.519, M05.521, M05.522, M05.529, M05.531, M05.532, M05.539, M05.541, M05.542, M05.549, M05.551, M05.552, M05.559, M05.561, M05.562, M05.569, M05.571, M05.572, M05.579, M05.59, M05.60, M05.611, M05.612, M05.619, M05.621, M05.622, M05.629, M05.631, M05.632, M05.639, M05.641, M05.642, M05.649, M05.651, M05.652, M05.659, M05.661, M05.662, M05.669, M05.671, M05.672, M05.679, M05.69, M05.70, M05.711, M05.712, M05.719, M05.721, M05.722, M05.729, M05.731, M05.732, M05.739, M05.741, M05.742, M05.749, M05.751, M05.752, M05.759, M05.761, M05.762, M05.769, M05.771, M05.772, M05.779, M05.79, M05.7A, M05.80, M05.811, M05.812, M05.819, M05.821, M05.822, M05.829, M05.831, M05.832, M05.839, M05.841, M05.842, M05.849, M05.851, M05.852, M05.859, M05.861, M05.862, M05.869, M05.871, M05.872, M05.879, M05.89, M05.8A, M05.9, M06.00, M06.011, M06.012, M06.019, M06.021, M06.022, M06.029, M06.031, M06.032, M06.039, M06.041, M06.042, M06.049, M06.051, M06.052, M06.059, M06.0
Combo of BIPL, PSDS, PSYCH_OTH, and SCHI (SMH)	F30.10, F30.11, F30.12, F30.13, F30.2, F30.3, F30.4, F30.8, F30.9, F31.0, F31.10, F31.11, F31.12, F31.13, F31.2, F31.30, F31.31, F31.32, F31.4, F31.5, F31.60, F31.61, F31.62, F31.63, F31.64, F31.70, F31.71, F31.72, F31.73, F31.74, F31.75, F31.76, F31.77, F31.78, F31.81, F31.89, F31.9, F33.8, F34.81, F34.89, F34.9, F39., F21., F34.0, F34.1, F60.0, F60.1, F60.2, F60.3, F60.4, F60.5, F60.6, F60.7, F60.81, F60.89, F60.9, F68.10, F68.11, F68.12, F68.13, F69., F06.0, F06.2, F22., F23., F24., F28., F29., F32.3, F33.3, F44.89, F20.0, F20.1, F20.2, F20.3, F20.5, F20.81, F20.89, F20.9, F25.0, F25.1, F25.8, F25.9

© 2025 Ferguson JM et al. *JAMA Network Open*.

Spinal Cord Injury (SPIINJ)	G96.11, S12.000A, S12.000B, S12.001A, S12.001B, S12.100A, S12.100B, S12.101A, S12.101B, S12.200A, S12.200B, S12.201A, S12.201B, S12.300A, S12.300B, S12.301A, S12.301B, S12.400A, S12.400B, S12.401A, S12.401B, S12.500A, S12.500B, S12.501A, S12.501B, S12.600A, S12.600B, S12.601A, S12.601B, S12.9XXA, S14.0XXA, S14.0XXS, S14.101A, S14.101S, S14.102A, S14.102S, S14.103A, S14.103S, S14.104A, S14.104S, S14.105A, S14.105S, S14.106A, S14.106S, S14.107A, S14.107S, S14.108A, S14.108S, S14.109A, S14.109S, S14.111A, S14.111S, S14.112A, S14.112S, S14.113A, S14.113S, S14.114A, S14.114S, S14.115A, S14.115S, S14.116A, S14.116S, S14.117A, S14.117S, S14.118A, S14.118S, S14.119A, S14.119S, S14.121A, S14.121S, S14.122A, S14.122S, S14.123A, S14.123S, S14.124A, S14.124S, S14.125A, S14.125S, S14.126A, S14.126S, S14.127A, S14.127S, S14.128A, S14.128S, S14.129A, S14.129S, S14.131A, S14.131S, S14.132A, S14.132S, S14.133A, S14.133S, S14.134A, S14.134S, S14.135A, S14.135S, S14.136A, S14.136S, S14.137A, S14.137S, S14.138A, S14.138S, S14.139A, S14.139S, S14.141A, S14.141S, S14.142A, S14.142S, S14.143A, S14.143S, S14.144A, S14.144S, S14.145A, S14.145S, S14.146A, S14.146S, S14.147A, S14.147S, S14.148A, S14.148S, S14.149A, S14.149S, S14.151A, S14.151S, S14.152A, S14.152S, S14.153A, S14.153S, S14.154A, S14.154S, S14.155A, S14.155S, S14.156A, S14.156S, S14.157A, S14.157S, S14.158A, S14.158S, S14.159A, S14.159S, S22.009A, S22.009B, S22.019A, S22.019B, S22.029A, S22.029B, S22.039A, S22.039B, S22.049A, S22.049B, S22.059A, S22.059B, S22.069A, S22.069B, S22.079A, S22.079B, S22.089A, S22.089B, S24.0XXA, S24.0XXS, S24.101A, S24.101S, S24.102A, S24.102S, S24.103A, S24.103S, S24.104A, S24.104S, S24.109A, S24.109S, S24.111A, S24.111S, S24.112A, S24.112S, S24.113A, S24.113S, S24.114A, S24.114S, S24.119A, S24.119S, S24.131A, S24.131S, S24.132A, S24.132S, S24.133A, S24.133S, S24.134A, S24.134S, S24.139A, S24.139S, S24.141A, S24.141S, S24.142A, S24.142S, S24.143A, S24.143S, S24.144A, S24.144S, S24.149A, S24.149S, S24.151A, S24.151S, S24.152A, S24.152S, S24.153A
Stroke/Transient Ischemic Attack (STROKE_TIA)	G45.0, G45.1, G45.2, G45.3, G45.8, G45.9, G46.0, G46.1, G46.2, G46.3, G46.4, G46.5, G46.6, G46.7, G46.8, G97.31, G97.32, I60.00, I60.01, I60.02, I60.10, I60.11, I60.12, I60.2, I60.20, I60.21, I60.22, I60.30, I60.31, I60.32, I60.4, I60.50, I60.51, I60.52, I60.6, I60.7, I60.8, I60.9, I61.0, I61.1, I61.2, I61.3, I61.4, I61.5, I61.6, I61.8, I61.9, I62.00, I62.01, I62.02, I62.9, I63.00, I63.011, I63.012, I63.013, I63.019, I63.02, I63.031, I63.032, I63.033, I63.039, I63.09, I63.10, I63.111, I63.112, I63.113, I63.119, I63.12, I63.131, I63.132, I63.133, I63.139, I63.19, I63.20, I63.211, I63.212, I63.213, I63.219, I63.22, I63.231, I63.232, I63.233, I63.239, I63.29, I63.30, I63.311, I63.312, I63.313, I63.319, I63.321, I63.322, I63.323, I63.329, I63.331, I63.332, I63.333, I63.339, I63.341, I63.342, I63.343, I63.349, I63.39, I63.40, I63.411, I63.412, I63.413, I63.419, I63.421, I63.422, I63.423, I63.429, I63.431, I63.432, I63.433, I63.439, I63.441, I63.442, I63.443, I63.449, I63.49, I63.50, I63.511, I63.512, I63.513, I63.519, I63.521, I63.522, I63.523, I63.529, I63.531, I63.532, I63.533, I63.539, I63.541, I63.542, I63.543, I63.549, I63.59, I63.6, I63.8, I63.81, I63.89, I63.9, I67.841, I67.848, I67.89, I97.810, I97.811, I97.820, I97.821
Combo of ALCO, DRUG_NonOUD, and OUDDx (SUD)	F10.10, F10.120, F10.121, F10.129, F10.130, F10.131, F10.132, F10.139, F10.14, F10.150, F10.151, F10.159, F10.180, F10.181, F10.182, F10.188, F10.19, F10.20, F10.220, F10.221, F10.229, F10.230, F10.231, F10.232, F10.239, F10.24, F10.250, F10.251, F10.259, F10.26, F10.27, F10.280, F10.281, F10.282, F10.288, F10.29, F10.920, F10.921, F10.929, F10.930, F10.931, F10.932, F10.939, F10.94, F10.950, F10.951, F10.959, F10.96, F10.97, F10.980, F10.981, F10.982, F10.988, F10.99, G62.1, I42.6, K29.20, K29.21, K70.0, K70.10, K70.11, K70.2, K70.30, K70.31, K70.40, K70.41, K70.9, P04.3, Q86.0, T51.0X1A, T51.0X2A, T51.0X3A, T51.0X4A, Z71.41, Z71.42, F11.13, F12.10, F12.120, F12.121, F12.122, F12.129, F12.13, F12.150, F12.151, F12.159, F12.180, F12.188, F12.19, F12.20, F12.220,

© 2025 Ferguson JM et al. *JAMA Network Open*.

	F12.221, F12.222, F12.229, F12.250, F12.251, F12.259, F12.280, F12.288, F12.29, F12.90, F12.920, F12.921, F12.922, F12.929, F12.950, F12.951, F12.959, F12.980, F12.988, F12.99, F13.10, F13.120, F13.121, F13.129, F13.130, F13.131, F13.132, F13.139, F13.14, F13.150, F13.151, F13.159, F13.180, F13.181, F13.182, F13.188, F13.19, F13.20, F13.220, F13.221, F13.229, F13.230, F13.231, F13.232, F13.239, F13.24, F13.250, F13.251, F13.259, F13.26, F13.27, F13.280, F13.281, F13.282, F13.288, F13.29, F13.90, F13.920, F13.921, F13.929, F13.930, F13.931, F13.932, F13.939, F13.94, F13.950, F13.951, F13.959, F13.96, F13.97, F13.980, F13.981, F13.982, F13.988, F13.99, F14.10, F14.120, F14.121, F14.122, F14.129, F14.13, F14.14, F14.150, F14.151, F14.159, F14.180, F14.181, F14.182, F14.188, F14.19, F14.20, F14.220, F14.221, F14.222, F14.229, F14.23, F14.24, F14.250, F14.251, F14.259, F14.280, F14.281, F14.282, F14.288, F14.29, F14.90, F14.920, F14.921, F14.922, F14.929, F14.93, F14.94, F14.950, F14.951, F14.959, F14.980, F14.981, F14.982, F14.988, F14.99, F15.10, F15.120, F15.121, F15.122, F15.129, F15.13, F15.14, F15.150, F15.151, F15.159, F15.180, F15.181, F15.182, F15.188, F15.19, F15.20, F15.220, F15.221, F15.222, F15.229, F15.23, F15.24, F15.250, F15.251, F15.259, F15.280, F15.281, F15.282, F15.288, F15.29, F15.90, F15.920, F15.921, F15.922, F15.929, F15.93, F15.94, F15.950, F15.951, F15.959, F15.980, F15.981, F15.982, F15.988, F15.99, F16.10, F16.120, F16.121, F16.122, F16.129, F16.14, F16.150, F16.151, F16.159, F16.180, F16.183, F16.188, F16.19, F16.20, F16.220, F16.221, F16.229, F16.24, F16.250, F16.251, F16.259, F16.280, F16.283, F16.288, F16.29, F16.90, F16.920, F16.921, F16.929, F16.94, F16.950, F16.951, F16.959, F16.980, F16.983, F16.988, F16.99, F18.10, F18.120, F18.121, F18.129, F18.14, F18.150, F18.151, F18.159, F18.17, F18.180, F18.188, F18.19, F18.20, F18.220, F18.221, F18.229, F18.24, F1, F11.10, F11.120, F11.121, F11.122, F11.129, F11.14, F11.150, F11.151, F11.159, F11.181, F11.182, F11.188, F11.19, F11.20, F11.220, F11.221, F11.222, F11.229, F11.23, F11.24, F11.250, F11.251, F11.259, F11.281, F11.282, F11.288, F11.29, F11.90, F11.920, F11.921, F11.922, F11.929, F11.93, F11.94, F11.950, F11.951, F11.959, F11.981, F11.982, F11.988, F11.99, T40.0X1A, T40.0X2A, T40.0X3A, T40.0X4A, T40.1X1A, T40.1X2A, T40.1X3A, T40.1X4A, T40.2X1A, T40.2X2A, T40.2X3A, T40.2X4A, T40.3X1A, T40.3X2A, T40.3X3A, T40.3X4A, T40.3X5A, T40.411A, T40.412A, T40.413A, T40.414A, T40.415A, T40.421A, T40.422A, T40.423A, T40.424A, T40.425A, T40.491A, T40.492A, T40.493A, T40.494A, T40.495A, T40.4X1A, T40.4X2A, T40.4X3A, T40.4X4A, T40.601A, T40.602A, T40.603A, T40.604A, T40.691A, T40.692A, T40.693A, T40.694A
Pressure and Chronic Ulcers (ULCERS)	I70.231, I70.232, I70.233, I70.234, I70.235, I70.238, I70.239, I70.241, I70.242, I70.243, I70.244, I70.245, I70.248, I70.249, I70.25, I70.331, I70.332, I70.333, I70.334, I70.335, I70.338, I70.339, I70.341, I70.342, I70.343, I70.344, I70.345, I70.348, I70.349, I70.35, I70.431, I70.432, I70.433, I70.434, I70.435, I70.438, I70.439, I70.441, I70.442, I70.443, I70.444, I70.445, I70.448, I70.449, I70.45, I70.531, I70.532, I70.533, I70.534, I70.535, I70.538, I70.539, I70.541, I70.542, I70.543, I70.544, I70.545, I70.548, I70.549, I70.55, I70.631, I70.632, I70.633, I70.634, I70.635, I70.638, I70.639, I70.641, I70.642, I70.643, I70.644, I70.645, I70.648, I70.649, I70.65, I70.731, I70.732, I70.733, I70.734, I70.735, I70.738, I70.739, I70.741, I70.742, I70.743, I70.744, I70.745, I70.748, I70.749, I70.75, L89.000, L89.001, L89.002, L89.003, L89.004, L89.006, L89.009, L89.010, L89.011, L89.012, L89.013, L89.014, L89.016, L89.019, L89.020, L89.021, L89.022, L89.023, L89.024, L89.026, L89.029, L89.100, L89.101, L89.102, L89.103, L89.104, L89.106, L89.109, L89.110, L89.111, L89.112, L89.113, L89.114, L89.116, L89.119, L89.120, L89.121, L89.122, L89.123, L89.124, L89.126, L89.129, L89.130, L89.131, L89.132, L89.133, L89.134, L89.136, L89.139, L89.140, L89.141, L89.142, L89.143, L89.144, L89.146, L89.149, L89.150, L89.151, L89.152, L89.153, L89.154, L89.156, L89.159, L89.200, L89.201, L89.202, L89.203, L89.204, L89.206, L89.209, L89.210, L89.211, L89.212, L89.213, L89.214, L89.216, L89.219, L89.220, L89.221, L89.222,

© 2025 Ferguson JM et al. *JAMA Network Open*.

	L89.223, L89.224, L89.226, L89.229, L89.300, L89.301, L89.302, L89.303, L89.304, L89.306, L89.309, L89.310, L89.311, L89.312, L89.313, L89.314, L89.316, L89.319, L89.320, L89.321, L89.322, L89.323, L89.324, L89.326, L89.329, L89.40, L89.41, L89.42, L89.43, L89.44, L89.45, L89.46, L89.500, L89.501, L89.502, L89.503, L89.504, L89.506, L89.509, L89.510, L89.511, L89.512, L89.513, L89.514, L89.516, L89.519, L89.520, L89.521, L89.522, L89.523, L89.524, L89.526, L89.529, L89.600, L89.601, L89.602, L89.603, L89.604, L89.606
Combo of CATARACT, GLAUCOMA_EVER, and VISUAL (VIS_IMP)	E08.36, E09.36, E10.36, E11.36, E13.36, H25.011, H25.012, H25.013, H25.019, H25.031, H25.032, H25.033, H25.039, H25.041, H25.042, H25.043, H25.049, H25.091, H25.092, H25.093, H25.099, H25.10, H25.11, H25.12, H25.13, H25.20, H25.21, H25.22, H25.23, H25.811, H25.812, H25.813, H25.819, H25.89, H25.9, H26.001, H26.002, H26.003, H26.009, H26.011, H26.012, H26.013, H26.019, H26.031, H26.032, H26.033, H26.039, H26.041, H26.042, H26.043, H26.049, H26.051, H26.052, H26.053, H26.059, H26.061, H26.062, H26.063, H26.069, H26.09, H26.101, H26.102, H26.103, H26.109, H26.111, H26.112, H26.113, H26.119, H26.121, H26.122, H26.123, H26.129, H26.131, H26.132, H26.133, H26.139, H26.20, H26.211, H26.212, H26.213, H26.219, H26.221, H26.222, H26.223, H26.229, H26.30, H26.31, H26.32, H26.33, H26.40, H26.411, H26.412, H26.413, H26.419, H26.491, H26.492, H26.493, H26.499, H26.8, H26.9, Q12.0, H40.011, H40.012, H40.013, H40.019, H40.021, H40.022, H40.023, H40.029, H40.041, H40.042, H40.043, H40.049, H40.051, H40.052, H40.053, H40.059, H40.10X0, H40.10X1, H40.10X2, H40.10X3, H40.10X4, H40.1110, H40.1111, H40.1112, H40.1113, H40.1114, H40.1120, H40.1121, H40.1122, H40.1123, H40.1124, H40.1130, H40.1131, H40.1132, H40.1133, H40.1134, H40.1190, H40.1191, H40.1192, H40.1193, H40.1194, H40.11X0, H40.11X1, H40.11X2, H40.11X3, H40.11X4, H40.1210, H40.1211, H40.1212, H40.1213, H40.1214, H40.1220, H40.1221, H40.1222, H40.1223, H40.1224, H40.1230, H40.1231, H40.1232, H40.1233, H40.1234, H40.1290, H40.1291, H40.1292, H40.1293, H40.1294, H40.1310, H40.1311, H40.1312, H40.1313, H40.1314, H40.1320, H40.1321, H40.1322, H40.1323, H40.1324, H40.1330, H40.1331, H40.1332, H40.1333, H40.1334, H40.1390, H40.1391, H40.1392, H40.1393, H40.1394, H40.1410, H40.1411, H40.1412, H40.1413, H40.1414, H40.1420, H40.1421, H40.1422, H40.1423, H40.1424, H40.1430, H40.1431, H40.1432, H40.1433, H40.1434, H40.1490, H40.1491, H40.1492, H40.1493, H40.1494, H40.151, H40.152, H40.153, H40.159, H40.20X0, H40.20X1, H40.20X2, H40.20X3, H40.20X4, H40.211, H40.212, H40.213, H40.219, H40.2210, H40.2211, H40.2212, H40.2213, H40.2214, H40.2220, H40.2221, H40.2222, H40.2223, H40.2224, H40.2230, H40.2231, H40.2232, H40.2233, H40.2234, H40.2290, H40.2291, H40.2292, H40.2293, H40.2294, H40.231, H40.232, H40.233, H40.239, H40.241, H40.242, H40.243, H40.249, H40.30X0, H40.30X1, H40.30X2, H40.30X3, H40.30X4, H40.31X0, H40.31X1, H40.31X2, H40.31X3, H40.31X4, H40.32X0, H40.32X1, H40.32X2, H40.32X3, H40.32X4, H40.33X0, H40.33X1, H40.33X2, H40.33X3, H40.33X4, H40.40X0, H40.40X1, H40.40X2, H40.40X3, H40.40X4, H40.41X0, H40.41X1, H40.41X2, H40.41X3, H40.41X4, H40.42X0, H40.42X1, H40.42X2, H40.42X3, H40.42X4, H40.43X0, H40.43X1, H40.43X2, H40.43X3, H40.43X4, H40.50X0, H40.50X1, H40.50X2, H40.50X3, H40.50X4, H40.51X0, H40.51X1, H40.51X2, H40.51X3, H40.51X4, H40.52X0,

© 2025 Ferguson JM et al. *JAMA Network Open*.

	H40.52X1, H40.52X2, H40.52X3, H40.52X4, H40.53X0, H40.53X1, H40.53X2, H40.53X3, H40.53X4, H40.60X0, H54.0, H54.0X33, H54.0X34, H54.0X35, H54.0X43, H54.0X44, H54.0X45, H54.0X53, H54.0X54, H54.0X55, H54.10, H54.11, H54.1131, H54.1132, H54.1141, H54.1142, H54.1151, H54.1152, H54.12, H54.1213, H54.1214, H54.1215, H54.1223, H54.1224, H54.1225, H54.2, H54.2X11, H54.2X12, H54.2X21, H54.2X22, H54.3, H54.8

* Conditions derived from Chronic Conditions Warehouse (CCW) unless noted otherwise.

** From H-CUP CLINICAL CLASSIFICATIONS SOFTWARE REFINED (CCSR) FOR ICD-10-CM DIAGNOSES, v2022.1

© 2025 Ferguson JM et al. *JAMA Network Open*.

eTable 4: Frequency of Veteran characteristics by 3,975,328 Veterans and their corresponding 7,144,371 outpatient primary care encounters occurring between April 1, 2022- March 31- 2023 at the Veterans Health Administration

	Unique Veterans*		Encounters							
			All		In-Person		Phone		Video	
	N=3,975,328		N=7,144,371		N=5,940,520		N=789,795		N=414,056	
	N	Col %	N	Col %	N	Col %	N	Col %	N	Col %
**Age category**										
18-44	589,859	14.8%	716,405	12.1%	98,307	12.4%	98,176	23.7%	716,405	12.1%
45-64	1,203,436	30.3%	1,781,135	30.0%	233,701	29.6%	161,697	39.1%	1,781,135	30.0%
65-75	1,166,985	29.4%	1,869,947	31.5%	245,885	31.1%	91,374	22.1%	1,869,947	31.5%
75-84	776,659	19.5%	1,223,987	20.6%	156,642	19.8%	47,388	11.4%	1,223,987	20.6%
85+	238,389	6.0%	349,046	5.9%	55,260	7.0%	15,421	3.7%	349,046	5.9%
**Race/Ethnicity**										
American Indian or Alaska Native	25,210	0.6%	37,479	0.6%	5,869	0.7%	2,160	0.5%	37,479	0.6%
Asian	45,489	1.1%	60,158	1.0%	11,178	1.4%	7,360	1.8%	60,158	1.0%
Black or African American	736,960	18.5%	1,157,632	19.5%	153,207	19.4%	96,923	23.4%	1,157,632	19.5%
Hispanic or Latino	286,914	7.2%	426,210	7.2%	66,536	8.4%	40,002	9.7%	426,210	7.2%
More than one race	32,240	0.8%	47,230	0.8%	7,528	1.0%	4,243	1.0%	47,230	0.8%
Native Hawaiian or Other Pacific Islander	30,379	0.8%	44,177	0.7%	8,495	1.1%	4,171	1.0%	44,177	0.7%
Unknown	251,961	6.3%	352,306	5.9%	53,498	6.8%	28,724	6.9%	352,306	5.9%
White	2,566,175	64.6%	3,815,328	64.2%	483,484	61.2%	230,473	55.7%	3,815,328	64.2%
**Gender**										
Male	3,582,876	90.1%	5,381,330	90.6%	702,737	89.0%	339,984	82.1%	5,381,330	90.6%
Female	382,885	9.6%	545,012	9.2%	84,883	10.7%	72,095	17.4%	545,012	9.2%
Gender Diverse	9,567	0.2%	14,178	0.2%	2,175	0.3%	1,977	0.5%	14,178	0.2%
**Rural/Urban Status**										
Urban	2,623,385	66.0%	3,914,021	65.9%	526,446	66.7%	316,114	76.3%	3,914,021	65.9%
Rural	1,206,096	30.3%	1,810,476	30.5%	234,538	29.7%	89,666	21.7%	1,810,476	30.5%
Highly Rural/Island	145,847	3.7%	216,023	3.6%	28,811	3.6%	8,276	2.0%	216,023	3.6%
**Marital Status**										
Married	2,270,143	57.1%	3,368,745	56.7%	423,233	53.6%	222,902	53.8%	3,368,745	56.7%
Widowed	160,282	4.0%	258,971	4.4%	36,254	4.6%	12,047	2.9%	258,971	4.4%
Separated/Divorced	972,122	24.5%	1,509,209	25.4%	216,033	27.4%	106,127	25.6%	1,509,209	25.4%
Single/Never Married	511,813	12.9%	725,247	12.2%	103,719	13.1%	65,365	15.8%	725,247	12.2%
Unknown/Missing	60,968	1.5%	78,348	1.3%	10,556	1.3%	7,615	1.8%	78,348	1.3%
**VHA Enrollment Priority Group**										
Group 1: >50% service-connected disability, Medal of Honor	1,662,136	41.8%	2,528,142	42.6%	354,011	44.8%	215,623	52.1%	2,528,142	42.6%
Group 2: 30-40% service-connected disability	316,141	8.0%	460,583	7.8%	55,880	7.1%	32,201	7.8%	460,583	7.8%
Group 3:1 0-20% service-connected disability, Prisoner of War, Purple Heart Medal, Discharged due to disability	510,806	12.8%	744,856	12.5%	89,879	11.4%	45,773	11.1%	744,856	12.5%
Group 4: VA catastrophically disabled, receive VA Aid	60,413	1.5%	95,635	1.6%	23,893	3.0%	6,386	1.5%	95,635	1.6%

© 2025 Ferguson JM et al. *JAMA Network Open*.

Group 5: Low income, receiving VA pension benefits	614,065	15.4%	967,136	16.3%	136,323	17.3%	50,904	12.3%	967,136	16.3%
Group 6: Military toxic exposures/recent combat; 0% service-connected disability	159,036	4.0%	215,488	3.6%	21,643	2.7%	11,658	2.8%	215,488	3.6%
Group 7: 0% service-connected disability, low-income, pays co-pays	126,228	3.2%	183,668	3.1%	24,481	3.1%	11,456	2.8%	183,668	3.1%
Group 8: No special enrollment considerations, pays co-pays	526,503	13.2%	745,012	12.5%	83,685	10.6%	40,055	9.7%	745,012	12.5%
**Drive time to VHA Primary Care**										
<5 minutes	170,721	4.3%	266,132	4.5%	35,802	4.5%	17,021	4.1%	266,132	4.5%
>5-11 minutes	805,092	20.3%	1,227,081	20.7%	161,668	20.5%	88,439	21.4%	1,227,081	20.7%
>11-17 minutes	969,238	24.4%	1,451,609	24.4%	190,496	24.1%	112,370	27.1%	1,451,609	24.4%
>17-25 minutes	903,641	22.7%	1,337,596	22.5%	178,380	22.6%	99,076	23.9%	1,337,596	22.5%
>25-48 minutes	914,998	23.0%	1,348,360	22.7%	178,966	22.7%	81,993	19.8%	1,348,360	22.7%
>48 minutes	211,638	5.3%	309,742	5.2%	44,483	5.6%	15,157	3.7%	309,742	5.2%
**VHA Facility Complexity**										
1a-High Complexity	1,848,600	46.5%	2,700,039	45.5%	398,093	50.4%	232,359	56.1%	2,700,039	45.5%
1b-High Complexity	1,005,253	25.3%	1,548,397	26.1%	179,872	22.8%	98,196	23.7%	1,548,397	26.1%
1c-High Complexity	529,721	13.3%	812,041	13.7%	97,371	12.3%	42,267	10.2%	812,041	13.7%
2-Medium Complexity	259,740	6.5%	388,962	6.5%	49,938	6.3%	16,159	3.9%	388,962	6.5%
3-Low Complexity	332,014	8.4%	491,081	8.3%	64,521	8.2%	25,075	6.1%	491,081	8.3%

Veterans could have more than one encounter in the study period.

© 2025 Ferguson JM et al. *JAMA Network Open*.

eTable 5: Risk ratios, adjusted mean probability, and the difference in the adjusted mean probability for **video-based care** by encounter diagnosis among 7,144,371 outpatient primary care encounters at the Veterans Health Administration occurring between April 1, 2022- March 31- 2023.

	Incidence Rate Ratio (95% CI)		Adjusted mean probability (95%CI)		Percentage Point difference in the adjusted mean probability (95%CI)	
Acute Myocardial Infarction	0.96	(0.88,1.05)	5.6%	(5.10,6.06)	-0.2%	(-0.69,0.27)
Anemia	0.95	(0.93,0.96)	5.5%	(5.43,5.59)	-0.3%	(-0.38,-0.22)
Anxiety/Depression/PTSD	0.99	(0.98,0.99)	5.7%	(5.69,5.77)	-0.1%	(-0.13,-0.04)
Asthma	0.94	(0.93,0.96)	5.5%	(5.38,5.56)	-0.3%	(-0.43,-0.24)
Atrial Fibrillation	0.94	(0.92,0.95)	5.4%	(5.35,5.54)	-0.4%	(-0.47,-0.27)
Benign Prostatic Hyperplasia	0.88	(0.87,0.90)	5.2%	(5.08,5.23)	-0.7%	(-0.76,-0.61)
Cancer	0.92	(0.90,0.93)	5.3%	(5.26,5.43)	-0.5%	(-0.56,-0.38)
Chronic Kidney Disease	0.93	(0.91,0.94)	5.4%	(5.31,5.47)	-0.4%	(-0.52,-0.35)
COPD	0.86	(0.85,0.88)	5.0%	(4.96,5.12)	-0.8%	(-0.89,-0.72)
Deafness and Hearing Impairment	0.78	(0.76,0.80)	4.5%	(4.45,4.65)	-1.3%	(-1.38,-1.18)
Dementia	1.86	(1.81,1.90)	10.7%	(10.39,10.93)	4.9%	(4.65,5.19)
Diabetes	0.92	(0.91,0.93)	5.4%	(5.39,5.47)	-0.5%	(-0.53,-0.43)
Epilepsy	0.96	(0.93,1.00)	5.6%	(5.37,5.79)	-0.2%	(-0.43,-0.01)
Fibromyalgia	1.08	(1.07,1.10)	6.2%	(6.15,6.30)	0.5%	(0.39,0.55)
Heart Failure	1.04	(1.01,1.06)	6.0%	(5.86,6.14)	0.2%	(0.07,0.35)
HIV/AIDS	1.52	(1.45,1.59)	8.8%	(8.41,9.18)	3.0%	(2.62,3.40)
Hyperlipidemia	0.81	(0.80,0.81)	5.1%	(5.03,5.08)	-1.2%	(-1.26,-1.18)
Hypertension	0.91	(0.90,0.91)	5.5%	(5.45,5.52)	-0.6%	(-0.60,-0.51)
Hypothyroidism	0.90	(0.89,0.91)	5.2%	(5.17,5.32)	-0.6%	(-0.67,-0.51)
Ischemic Heart Disease	0.88	(0.87,0.90)	5.2%	(5.09,5.23)	-0.7%	(-0.76,-0.61)
Liver Disease/Cirrhosis	1.03	(1.01,1.05)	5.9%	(5.82,6.07)	0.2%	(0.03,0.28)
Lower Back Pain	0.90	(0.89,0.91)	5.3%	(5.27,5.36)	-0.6%	(-0.65,-0.56)
Migraine	1.01	(0.99,1.02)	5.8%	(5.76,5.92)	0.0%	(-0.03,0.13)
Mobility Impairment	1.25	(1.18,1.32)	7.2%	(6.84,7.61)	1.4%	(1.04,1.82)
Multiple Sclerosis	1.10	(1.04,1.16)	6.4%	(6.00,6.73)	0.6%	(0.21,0.93)
Obesity	0.86	(0.85,0.87)	5.1%	(5.00,5.11)	-0.8%	(-0.89,-0.77)
Osteoporosis	0.91	(0.87,0.95)	5.3%	(5.04,5.50)	-0.5%	(-0.76,-0.30)
Parkinson's Disease	1.29	(1.24,1.34)	7.5%	(7.20,7.75)	1.7%	(1.41,1.97)
Peripheral Vascular Disease	0.84	(0.81,0.87)	4.9%	(4.73,5.07)	-0.9%	(-1.08,-0.73)
Pneumonia	0.95	(0.90,1.00)	5.5%	(5.19,5.80)	-0.3%	(-0.61,0.00)
Pressure and Chronic Ulcers	1.37	(1.29,1.46)	8.0%	(7.46,8.44)	2.2%	(1.67,2.65)
Rheumatoid Arthritis/Osteoarthritis	0.83	(0.82,0.84)	4.9%	(4.82,4.95)	-1.0%	(-1.05,-0.92)
Severe Mental Health Disorders	0.93	(0.92,0.95)	5.4%	(5.33,5.52)	-0.4%	(-0.49,-0.29)
Spinal Cord Injury	1.02	(0.85,1.23)	5.9%	(4.82,7.01)	0.1%	(-0.98,1.22)
Stroke/Transient Ischemic Attack	1.09	(1.06,1.12)	6.3%	(6.14,6.48)	0.5%	(0.36,0.70)
Substance Use Disorders	0.84	(0.82,0.85)	4.9%	(4.82,4.98)	-0.9%	(-1.03,-0.86)
Traumatic Brain Injury	1.16	(1.06,1.27)	6.7%	(6.12,7.35)	0.9%	(0.32,1.55)
Viral Hepatitis	0.86	(0.82,0.90)	5.0%	(4.77,5.22)	-0.8%	(-1.03,-0.58)

© 2025 Ferguson JM et al. *JAMA Network Open*.

Visual Impairments	0.89	(0.87,0.93)	5.2%	(5.02,5.37)	-0.6%	(-0.79,-0.44)

Abbreviations: COPD: chronic obstructive pulmonary disease, PTSD: Post-traumatic stress disorder
Model was adjusted age, sex, race, ethnicity, rurality, marital status, VHA enrollment priority group, drive time from a primary care facility) and other chronic conditions managed during the encounter.

© 2025 Ferguson JM et al. *JAMA Network Open*.

eTable 6: Risk ratios, adjusted mean probability, and the difference in the adjusted mean probability for **phone-based care** by encounter diagnosis among 7,144,371 outpatient primary care encounters at the Veterans Health Administration occurring between April 1, 2022- March 31- 2023

	Incidence Rate Ratio (95% CI)		Adjusted mean probability (95%CI)		Percentage Point difference in the adjusted mean probability (95%CI)	
Acute Myocardial Infarction	0.94	(0.89,0.99)	10.4%	(9.88,10.96)	-0.6%	(-1.18,-0.10)
Anemia	0.91	(0.90,0.91)	10.1%	(9.97,10.17)	-1.1%	(-1.15,-0.95)
Anxiety/Depression/PTSD	0.70	(0.70,0.71)	8.3%	(8.22,8.33)	-3.5%	(-3.56,-3.44)
Asthma	0.71	(0.70,0.72)	7.9%	(7.79,8.04)	-3.2%	(-3.37,-3.12)
Atrial Fibrillation	0.74	(0.74,0.75)	8.4%	(8.27,8.46)	-2.9%	(-2.98,-2.78)
Benign Prostatic Hyperplasia	0.70	(0.69,0.71)	7.9%	(7.83,7.99)	-3.4%	(-3.49,-3.32)
Cancer	0.77	(0.76,0.78)	8.6%	(8.53,8.72)	-2.6%	(-2.70,-2.50)
Chronic Kidney Disease	0.94	(0.93,0.95)	10.4%	(10.33,10.54)	-0.7%	(-0.77,-0.56)
COPD	0.81	(0.80,0.81)	9.0%	(8.96,9.13)	-2.2%	(-2.28,-2.10)
Deafness and Hearing Impairment	0.54	(0.53,0.55)	6.1%	(5.95,6.15)	-5.2%	(-5.29,-5.08)
Dementia	1.01	(0.99,1.03)	11.2%	(10.99,11.41)	0.2%	(-0.06,0.36)
Diabetes	0.90	(0.89,0.90)	10.2%	(10.14,10.25)	-1.2%	(-1.22,-1.10)
Epilepsy	0.80	(0.78,0.82)	8.8%	(8.59,9.10)	-2.2%	(-2.49,-1.97)
Fibromyalgia	0.95	(0.95,0.96)	10.6%	(10.49,10.68)	-0.5%	(-0.61,-0.41)
Heart Failure	1.09	(1.07,1.10)	12.0%	(11.83,12.17)	1.0%	(0.81,1.15)
HIV/AIDS	1.24	(1.19,1.28)	13.7%	(13.18,14.15)	2.6%	(2.13,3.10)
Hyperlipidemia	0.59	(0.59,0.59)	7.8%	(7.72,7.79)	-5.4%	(-5.48,-5.37)
Hypertension	0.55	(0.54,0.55)	7.8%	(7.81,7.87)	-6.5%	(-6.53,-6.41)
Hypothyroidism	0.79	(0.78,0.79)	8.8%	(8.71,8.89)	-2.4%	(-2.51,-2.32)
Ischemic Heart Disease	0.76	(0.75,0.76)	8.6%	(8.50,8.65)	-2.7%	(-2.82,-2.66)
Liver Disease/Cirrhosis	0.99	(0.97,1.01)	10.9%	(10.78,11.12)	-0.1%	(-0.28,0.06)
Lower Back Pain	0.75	(0.75,0.76)	8.7%	(8.69,8.80)	-2.9%	(-2.92,-2.80)
Migraine	0.74	(0.73,0.75)	8.2%	(8.11,8.33)	-3.0%	(-3.07,-2.84)
Mobility Impairment	1.21	(1.16,1.26)	13.3%	(12.82,13.85)	2.3%	(1.77,2.81)
Multiple Sclerosis	0.96	(0.91,1.01)	10.6%	(10.07,11.19)	-0.4%	(-0.98,0.13)
Obesity	0.62	(0.61,0.63)	7.1%	(7.00,7.14)	-4.3%	(-4.43,-4.27)
Osteoporosis	0.83	(0.80,0.85)	9.1%	(8.88,9.41)	-1.9%	(-2.19,-1.66)
Parkinson's Disease	0.87	(0.84,0.89)	9.6%	(9.35,9.89)	-1.5%	(-1.73,-1.18)
Peripheral Vascular Disease	0.85	(0.84,0.87)	9.5%	(9.27,9.66)	-1.6%	(-1.81,-1.42)
Pneumonia	0.95	(0.93,0.98)	10.5%	(10.24,10.84)	-0.5%	(-0.82,-0.22)
Pressure and Chronic Ulcers	0.93	(0.89,0.98)	10.3%	(9.88,10.78)	-0.7%	(-1.18,-0.27)
Rheumatoid Arthritis/Osteoarthritis	0.73	(0.72,0.73)	8.2%	(8.15,8.30)	-3.1%	(-3.18,-3.02)
Severe Mental Health Disorders	0.77	(0.76,0.78)	8.6%	(8.46,8.73)	-2.5%	(-2.68,-2.40)
Spinal Cord Injury	1.16	(1.06,1.28)	12.8%	(11.62,14.05)	1.8%	(0.57,2.99)
Stroke/Transient Ischemic Attack	0.97	(0.95,0.98)	10.7%	(10.48,10.88)	-0.4%	(-0.59,-0.18)
Substance Use Disorders	0.76	(0.75,0.77)	8.5%	(8.41,8.63)	-2.7%	(-2.77,-2.54)
Traumatic Brain Injury	0.78	(0.72,0.85)	8.6%	(7.88,9.31)	-2.5%	(-3.18,-1.74)
Viral Hepatitis	0.81	(0.79,0.84)	9.0%	(8.72,9.29)	-2.1%	(-2.35,-1.78)
Visual Impairments	0.77	(0.75,0.79)	8.5%	(8.31,8.70)	-2.6%	(-2.78,-2.39)

© 2025 Ferguson JM et al. *JAMA Network Open*.

Abbreviations: COPD: chronic obstructive pulmonary disease, PTSD: Post-traumatic stress disorder
Model was adjusted age, sex, race, ethnicity, rurality, marital status, VHA enrollment priority group, drive time from a primary care facility) and other chronic conditions managed during the encounter.

© 2025 Ferguson JM et al. *JAMA Network Open*.

eTable 7: Risk ratios, adjusted mean probability, and the difference in the adjusted mean probability for **in-person care** by encounter diagnosis among 7,144,371 outpatient primary care encounters at the Veterans Health Administration occurring between April 1, 2022- March 31- 2023

	Incidence Rate Ratio (95% CI)		Adjusted mean probability (95%CI)		Percentage Point difference in the adjusted mean probability (95%CI)	
Acute Myocardial Infarction	1.00	(1.00,1.01)	83.5%	(82.82,84.18)	0.4%	(-0.33,1.03)
Anemia	1.01	(1.01,1.01)	84.0%	(83.84,84.08)	0.9%	(0.74,0.98)
Anxiety/Depression/PTSD	1.04	(1.04,1.04)	85.8%	(85.71,85.85)	3.3%	(3.19,3.35)
Asthma	1.04	(1.04,1.04)	86.4%	(86.23,86.57)	3.4%	(3.18,3.52)
Atrial Fibrillation	1.04	(1.03,1.04)	85.9%	(85.79,86.01)	2.9%	(2.82,3.06)
Benign Prostatic Hyperplasia	1.04	(1.04,1.04)	86.1%	(86.01,86.18)	3.2%	(3.15,3.33)
Cancer	1.03	(1.03,1.03)	85.7%	(85.60,85.82)	2.7%	(2.63,2.86)
Chronic Kidney Disease	1.01	(1.01,1.01)	83.9%	(83.79,84.00)	0.8%	(0.70,0.92)
COPD	1.03	(1.03,1.03)	85.4%	(85.26,85.47)	2.4%	(2.29,2.51)
Deafness and Hearing Impairment	1.06	(1.06,1.06)	88.1%	(88.01,88.24)	5.2%	(5.07,5.30)
Dementia	0.94	(0.94,0.95)	78.4%	(78.06,78.71)	-4.8%	(-5.16,-4.52)
Diabetes	1.01	(1.01,1.01)	84.0%	(83.91,84.03)	1.1%	(1.08,1.22)
Epilepsy	1.03	(1.02,1.03)	85.5%	(85.15,85.85)	2.4%	(2.02,2.72)
Fibromyalgia	0.99	(0.99,0.99)	82.4%	(82.31,82.57)	-0.8%	(-0.90,-0.63)
Heart Failure	0.98	(0.98,0.98)	81.7%	(81.51,81.90)	-1.5%	(-1.70,-1.30)
HIV/AIDS	0.91	(0.90,0.91)	75.3%	(74.61,76.05)	-7.8%	(-8.57,-7.13)
Hyperlipidemia	1.08	(1.08,1.08)	86.7%	(86.63,86.72)	6.4%	(6.34,6.47)
Hypertension	1.09	(1.09,1.09)	86.5%	(86.44,86.52)	7.1%	(7.01,7.15)
Hypothyroidism	1.03	(1.03,1.03)	85.7%	(85.59,85.80)	2.7%	(2.63,2.85)
Ischemic Heart Disease	1.03	(1.03,1.03)	85.5%	(85.45,85.61)	2.7%	(2.59,2.77)
Liver Disease/Cirrhosis	1.00	(0.99,1.00)	82.8%	(82.61,83.00)	-0.4%	(-0.55,-0.16)
Lower Back Pain	1.04	(1.04,1.04)	85.7%	(85.66,85.82)	3.1%	(3.02,3.19)
Migraine	1.03	(1.03,1.03)	85.6%	(85.41,85.76)	2.5%	(2.35,2.70)
Mobility Impairment	0.93	(0.93,0.94)	77.7%	(76.94,78.42)	-5.5%	(-6.23,-4.75)
Multiple Sclerosis	0.98	(0.97,0.99)	81.7%	(80.83,82.57)	-1.5%	(-2.32,-0.58)
Obesity	1.05	(1.05,1.05)	87.2%	(87.07,87.25)	4.4%	(4.34,4.53)
Osteoporosis	1.03	(1.03,1.04)	85.9%	(85.54,86.24)	2.8%	(2.41,3.11)
Parkinson's Disease	1.00	(0.99,1.00)	83.0%	(82.65,83.42)	-0.1%	(-0.50,0.27)
Peripheral Vascular Disease	1.02	(1.02,1.02)	85.0%	(84.75,85.17)	1.8%	(1.63,2.05)
Pneumonia	0.99	(0.98,0.99)	82.0%	(81.47,82.60)	-1.1%	(-1.68,-0.55)
Pressure and Chronic Ulcers	0.97	(0.96,0.97)	80.2%	(79.42,81.07)	-2.9%	(-3.74,-2.08)
Rheumatoid Arthritis/Osteoarthritis	1.04	(1.04,1.04)	86.4%	(86.28,86.46)	3.5%	(3.43,3.63)
Severe Mental Health Disorders	1.03	(1.03,1.04)	85.9%	(85.72,86.08)	2.8%	(2.66,3.02)
Spinal Cord Injury	0.94	(0.91,0.97)	78.1%	(75.79,80.42)	-5.0%	(-7.36,-2.74)
Stroke/Transient Ischemic Attack	1.00	(0.99,1.00)	82.8%	(82.61,83.09)	-0.3%	(-0.55,-0.07)
Substance Use Disorders	1.04	(1.04,1.04)	86.5%	(86.39,86.68)	3.5%	(3.38,3.68)
Traumatic Brain Injury	1.01	(1.00,1.02)	84.1%	(82.91,85.20)	0.9%	(-0.24,2.05)
Viral Hepatitis	1.04	(1.03,1.04)	86.0%	(85.69,86.40)	2.9%	(2.56,3.27)
Visual Impairments	1.03	(1.03,1.04)	85.8%	(85.58,86.02)	2.7%	(2.47,2.91)

© 2025 Ferguson JM et al. *JAMA Network Open*.

Abbreviations: COPD: chronic obstructive pulmonary disease, PTSD: Post-traumatic stress disorder
All models were adjusted age, sex, race, ethnicity, rurality, marital status, VHA enrollment priority group, drive time from a primary care facility) and other chronic conditions managed during the encounter.

© 2025 Ferguson JM et al. *JAMA Network Open*.
