# Supplementary material for: Variability in Primary Care Telehealth Delivery Methods Across Chronic Conditions
Source: JAMA Netw Open. 2025 Mar 26;8(3):e251988. doi: 10.1001/jamanetworkopen.2025.1988 (PMC11947845; doi:10.1001/jamanetworkopen.2025.1988)
Supplement: Supplement 2. — Data Sharing Statement [file jamanetwopen-e251988-s002.pdf]

Data Sharing Statement

Ferguson. Variability in Primary Care Telehealth Delivery Methods Across Chronic Conditions. *JAMA Netw Open*. Published March 26, 2025. doi:10.1001/jamanetworkopen.2025.1988

Data
Data available: No

Additional Information
Explanation for why data not available: Due to US Department of Veterans Affairs (VA) regulations and our ethics agreements, the analytic data used for this evaluation are not permitted to leave the VA firewall without a Data Use Agreement. VA data are made freely available to VA authorized researchers with an approved VA protocol. For more information, please visit https://www.virec.research.va.gov or contact the VA Information Resource Center at VIReC@va.gov
